# Disparities in COVID-19 vaccine uptake, attitudes, and experiences between food system and non–food system essential workers

**DOI:** 10.5304/jafscd.2024.132.012

**Published:** 2024

**Authors:** Brianna L. Smarsh, David Yankey, Mei-Chuan Hung, Heidi M. Blanck, Jennifer L. Kriss, Michael A. Flynn, Peng-Jun Lu, Sherri McGarry, Adrienne C. Eastlake, Alfonso Rodriguez Lainz, James A. Singleton, Jennifer M. Lincoln

**Affiliations:** a Division of Nutrition, Physical Activity, and Obesity, National Center for Chronic Disease Prevention and Health Promotion, Centers for Disease Control and Prevention, Atlanta, GA, USA.; b CDC COVID-19 Emergency Response, Atlanta, GA, USA.; c National Institute for Occupational Safety and Health, Centers for Disease Control and Prevention, Cincinnati, OH, USA.; d Immunization Services Division, National Center for Immunization and Respiratory Diseases, Centers for Disease Control and Prevention, Atlanta, GA, USA.; e Division of Foodborne, Waterborne, and Environmental Diseases, National Center for Emerging and Zoonotic Infectious Diseases, Atlanta, GA, USA.; f Division of Global Migration and Quarantine, National Center for Emerging and Zoonotic Infectious Diseases, CDC, Centers for Disease Control and Prevention, Atlanta, GA, USA.; g Leidos, Incorporated, Atlanta, Georgia.

**Keywords:** COVID-19, COVID-19 vaccine, essential workers, food system, food security, occupational health, agriculture workers, food workers, health equity, vaccine equity

## Abstract

The COVID-19 pandemic has disproportionately affected the health of food system (FS) essential workers compared with other essential and non-essential workers. Even greater disparity exists for workers in certain FS work settings and for certain FS worker subpopulations. We analyzed essential worker respondents (*n* = 151,789) in May–November 2021 data from the National Immunization Survey Adult COVID Module (NIS-ACM) to assess and characterize COVID-19 vaccination uptake (≥1 dose) and intent (reachable, reluctant), attitudes about COVID-19 and the vaccine, and experiences and difficulties getting the vaccine. We compared rates, overall and by certain characteristics, between workers of the same group, and between FS (*n* = 17,414) and non–food system (NFS) worker groups (*n* = 134,375), to determine if differences exist. FS worker groups were classified as “agriculture, forestry, fishing, or hunting” (AFFH; *n* = 2,730); “food manufacturing facility” (FMF; *n* = 3,495); and “food and beverage store” (FBS; *n* = 11,189). Compared with NFS workers, significantly lower percentages of FS workers reported ≥1 dose of COVID-19 vaccine or vaccine requirements at work or school, but overall vaccine experiences and difficulties among vaccinated FS workers were statistically similar to NFS workers. When we examined intent regarding COVID-19 vaccination among unvaccinated FS workers compared with NFS counterparts, we found a higher percentage of FMF and FBS workers were reachable whereas a higher percentage of AFFH workers were reluctant about vaccination, with differences by sociodemographic characteristics. Overall, results showed differences in uptake, intent, and attitudes between worker groups and by some sociodemographic characteristics. The findings reflect the diversity of FS workers and underscore the importance of collecting occupational data to assess health inequalities and of tailoring efforts to worker groups to improve confidence and uptake of vaccinations for infectious diseases such as COVID-19. The findings can inform future research, adult infectious disease interventions, and emergency management planning.

## Introduction

The Food and Agriculture Sector is one of 16 critical infrastructure sectors considered essential by the U.S. Cybersecurity and Infrastructure Security Agency for continuing critical infrastructure operations during emergencies, including the COVID-19 pandemic (Cybersecurity & Infrastructure Security Agency, 2020). This sector generally includes farming and food manufacturing, processing, and operating storage facilities, as well as operating retail food stores and restaurants. It accounts for 10.3% of total U.S. employment (19.7 million part- and full-time jobs) and 5.2% of U.S. gross domestic product (Kassel & Morrison, 2020).

Research demonstrates that a variety of factors can influence COVID-19 vaccine uptake, such as age, education level, health insurance status, work and school vaccine mandates, and attitudes or behaviors such as perceived efficacy of the vaccine and concern about getting sick with COVID-19 (Centers for Disease Control and Prevention, 2023a; Roy et al., 2022). Further, group traits can affect the actions and attitudes of members. The COVID-19 pandemic posed an increased occupational health risk to many essential workers; for instance, being unable to stay home during community shutdowns, inadequate personal protective equipment, and regular interactions with individuals of unknown COVID-19 status. But the increased risk was not experienced equally by all essential workers. In the case of food and agriculture workers (hereafter, food system workers, or FS workers), overlapping pandemic occupational vulnerabilities elevated risk, such as close proximity to fellow workers for long periods of time, work conditions with poor airflow and ventilation, riding to and from work in overcrowded buses or vans, and being exposed for prolonged periods to customers/the general public, including some who had to remove protective masks to eat and drink, or refused to comply with masking protocols in general. Structural barriers to mitigating FS worker risk included factors such as limited institutional capacity of organizations to support workers (e.g., funding, translation services) and logistical challenges (e.g., mobile nature of some FS jobs). FS worker health was known, pre-pandemic, to be disproportionately affected by the cumulative precarity resulting from overlapping vulnerabilities. These encompass the overrepresentation of racial and ethnic minorities, immigrants, and workers who are financially and socially vulnerable due to factors such as low pay, occupational exceptionalism,^1^ temporary or precarious job situations, shift work, immigration status, limited English proficiency, lack of health insurance, and discrimination and systemic racism (Dempsey et al., 2022; Fan & Pena, 2021; Flynn et al., 2014; Flynn, Cunningham et al., 2015; Flynn, Eggerth et al., 2015; Gelatt, 2020; Gravel & Dubé, 2016; Parks et al., 2020; Ramos et al., 2020; Rodman et al., 2016; Rolland & Kim, 2021; Sajjanhar & Mohammed, 2021; Thomas et al., 2021). These pre-pandemic and pandemic vulnerabilities have been extensively linked to increased and excessive morbidity and mortality among FS workers during the pandemic compared with some other essential and non-essential workers; the impacts were even greater for workers in certain FS work settings and for those from some racial/ethnic minority and immigrant groups (Billock et al., 2022; Bui et al., 2020; Chen et al., 2022; Cummings et al., 2022; Dyal et al., 2020; Hawkins, 2020; Lusk & Chandra, 2021; Obinna, 2021; Rubenstein et al., 2020; UCLA Labor Center, 2022; Waltenburg et al., 2021).

FS workers were a priority population for COVID-19 vaccination. On December 20, 2020, the Advisory Committee on Immunization Practices recommended prioritizing FS workers in Phase 1b (food and agricultural workers, grocery store workers, food manufacturing) and Phase 1c (food service workers) for COVID-19 vaccine allocation (Dooling et al., 2020). FS essential workers have been identified as a group of focus for achieving vaccine equity (CDC, 2020). Other studies have assessed vaccine uptake, intent to vaccinate, attitudes and perceptions toward the vaccine, and barriers to uptake among varying sectors of essential workers, particularly those in healthcare. To our knowledge, however, no large-scale COVID-19 vaccine-related studies or surveillance has focused solely on FS essential workers (Henneberger et al., 2022; King et al., 2021; Nguyen et al., 2021; Schneider et al., 2021; Steege et al., 2022).

Work is a social determinant of health. Collecting information about occupations and work settings facilitates improved understanding of the causes of health inequities, provides information to evaluate risks among various groups of workers, and helps refine guidance for specific industry and occupational groups (Ahonen et al., 2018; Flynn et al., 2022; Luckhaupt et al., 2020; Marovich et al., 2021; Silver et al., 2022). Recognizing these facts, as well as the information gaps related to COVID-19 status, intent, attitudes, and behaviors for FS essential workers, the objectives of this study were to describe and characterize COVID-19 vaccination status and intent, attitudes about the vaccine and COVID-19, and vaccine experiences, from April 22 through November 27, 2021 for three groups of FS workers in the U.S., and to compare differences between FS and non–food system (NFS) worker groups, and between workers in the same occupational group, to determine if disparities exist. Findings can inform the refinement of future analyses of these topics and groups, interventions for adult vaccination for infectious diseases, and planning for programmatic and policy aspects of future emergency management.

## Methods

Study data, measures, qualitative analysis (the inclusion of free-text responses) and statistical analysis are described below.

### Data

The National Immunization Survey-Adult COVID Module (NIS-ACM)^2^ is a random-digit-dialed cellular telephone survey of U.S. adults 18 years and older. Survey respondents were sampled within all 50 states and the District of Columbia, as well as selected local areas (Bexar County, Texas; Chicago, Illinois; Houston, Texas; New York, New York; and Philadelphia County, Pennsylvania) and U.S. territories (Guam [April–July 2021], Puerto Rico, and the U.S. Virgin Islands). Surveys were conducted in English and Spanish. Participants preferring another language were interviewed using contracted phone interpretation services (Language-LineSolutions, over 140 languages available).

Survey respondents from April 22 through November 27, 2021 (hereafter “May to November 2021”) who reported that they were a frontline or essential worker (hereafter referred to as “essential workers”) were included in the analysis (*n* = 151,789). Monthly survey response rates were calculated according to the American Association for Public Opinion Research type 3 response rate^3^ and ranged from 17.2% to 21.4%.

### Measures

NIS-ACM^4^ included questions about COVID-19 vaccination status and intent, attitudes and perceptions about COVID-19 vaccine, experiences getting a COVID-19 vaccine, sociodemographic characteristics, and essential worker status. Two questions assessed COVID-19 vaccination status and intent: “Have you received at least one dose of a COVID-19 vaccine?” and if not, “How likely are you to get a COVID-19 vaccine? Would you say you would definitely get a vaccine, probably get a vaccine, probably not get a vaccine, definitely not get a vaccine, or are not sure?” Those who reported having at least one dose were considered “vaccinated”; those who said they definitely will get vaccinated, probably will get vaccinated, or were unsure were considered “reachable”; and those who said they probably or definitely would not get vaccinated were considered “reluctant.” Three questions assessed respondents’ attitudes and perceptions about COVID-19 and the vaccine (*n* = 151,789), and five questions assessed experiences and difficulties getting the vaccine (*n* = 129,994); vaccination status/intent was not a prerequisite for questions about attitudes or experiences, and respondents could answer regardless of vaccination status. Outcomes related to experiences and difficulties getting the vaccine were stratified by vaccination status (vaccinated, unvaccinated).

Respondents self-reported their sex, race and ethnicity, age, household income, health insurance status, foreign-born status, comorbidity^5^ status (have any or none), and zip code or city of residence. Urbanicity, as defined by metropolitan statistical area (MSA) classification (MSA principal city, MSA non-principal city, and non-MSA), was determined based on household-reported city and county of residence (Office of Management and Budget, 2010). Household income was categorized relative to the U.S. Census Bureau 2020 poverty threshold and at the level of $75,000 (U.S. Census Bureau, 2022).

Essential worker status was self-reported and based on the questions “Are you a frontline or essential worker according to your state or region?” and “In what location or setting do you currently work*?*” Respondents who reported being a frontline or essential worker provided the interviewer with a work location or setting; then interviewers selected a grouping category from a predetermined list of 14 frontline/essential industry/occupation groups,^6^ or grouped the respondent in an “other” category if they could not be grouped in the existing list of 14. The list of 14 industry/occupation groups included FS categories: “agriculture, forestry, fishing, or hunting” (AFFH); “food manufacturing facility” (FMF); and “food and beverage store” (FBS). For those who selected the “other” category, interviewers entered a free-text response for the respondent’s self-reported occupation type or setting. Free-text responses are open-ended responses that allow respondents to answer in their own words; these qualitative data require additional analysis to summarize and organize to be useful.

### Inclusion of Free-text Responses

To assess whether free-text responses from respondents who answered “other” (*n* = 19,464) to the occupation location or setting question contained essential industries and occupations from the predetermined list in the survey questionnaire, we used the National Institute of Occupational Safety and Health (NIOSH) Industry and Occupation Computerized Coding System (NIOCCS), a web-based software tool designed to translate industry and occupation text to standardized industry and occupation codes (NIOSH, 2022). NIOCCS output produces an Excel file with titles and codes for four items: Census Industry, Census Occupation, North American Industry Classification System, and Standard Occupational Classification. Occupational title/codes from NIOCC output were manually reviewed for classification into one of our three FS groups (AFFH, FMF, or FBS), and remaining entries, such as education and health occupations, were assigned to the NFS worker group. Two authors completed two rounds of random 10% samples: 10% of entries from the total 19,464 sample (*n* = 1,946) and 10% of all entries that were assigned to a FS industry occupation group (*n* = 136). Discrepancies were discussed until group consensus could be reached on a final grouping determination. See Table 1 for results of the free-text analysis.

### Analysis

Weighted estimates and 95% confidence intervals (CIs) were generated for vaccination status and intent, vaccine attitudes and perceptions, and experiences getting COVID-19 vaccination. Respondents grouped in AFFH, FMF, or FBS were considered to be FS workers. The remaining industry occupation response options were considered NFS^7^ workers. All analyses were stratified by the three groups of FS workers (AFFH, FMF, FBS) and one group of NFS workers. T-tests for proportions tested for differences between workers within the same worker group^8^ and between FS and NFS workers,^9^ with *P* values <0.05 considered statistically significant. Data were weighted to represent the noninstitutionalized U.S. adult population and calibrated to state-level vaccine administration data reported to the Centers for Disease Control and Prevention (CDC, 2023a, 2023b). Analyses were conducted using SAS (version 9.4; SAS Institute) and SUDAAN (version 11; RTI International). CDC reviewed this activity, which was conducted consistently with applicable federal law and CDC policy (45 C.F.R. part 46, 21 C.F.R. part 56; 42 U.S.C. Sect. 241(d); 5 U.S.C. Sect. 552a; 44 U.S.C. Sect. 3501 et seq).

## Results

Below, we describe results for vaccination status and intent, attitudes and perceptions of COVID-19 and the COVID-19 vaccine, and experiences and difficulties with the vaccine.

### Vaccination Status and Intent

Results for three outcomes related to vaccination status and intent are described.

### Vaccinated (>1 dose)

Overall, uptake of ≥1 COVID-19 vaccine dose was significantly lower among all FS worker groups (AFFH 58.5%, FMF 59.8%, FBS 61.6%) compared to NFS worker groups (68.5%) (Table A1).

When assessed by sociodemographic characteristics within each worker group, coverage significantly differed for FS and NFS workers by race/ethnicity (higher coverage among Asian compared with referent non-Hispanic White (NH-White)), age (higher coverage among 40–49, 50–64, or 65+ compared with referent 18–29), health insurance status (lower coverage among uninsured compared with referent insured), urbanicity (lower coverage in non-MSA compared with referent principal city MSA), and month of interview. There were additional significant differences among workers with ≥1 dose within each of the three FS groups; for example, large differences among AFFH workers by comorbidity status, and statistical variation by FBS worker race/ethnicity groups (Hispanic, AI/AN) compared with NH-White workers not seen with other FS worker groups.

When FS worker groups were stratified by sociodemographic subgroups and compared with NFS counterparts, significantly lower percentages of FS workers who were NH-White, 30–39 years, insured, non-foreign born, without comorbidities, and residing in a non-principal city MSA or non-MSA reported having ≥1 dose (Table A1).

### Unvaccinated, Reachable

The overall percentage of reachable workers was significantly higher among FMF (18.9%) and FBS (20.4%) workers compared with NFS (13.3%) workers. When compared to workers in the same worker group, reachable FS and NFS workers significantly differed by race/ethnicity (but not consistently the same subgroups, compared with referent NH-White groups) and age (lower percentage of ages 40–49, 50–64, or 65+ reachable, compared with referent ages 18–29). Reachable FS workers did not statistically differ by sex or foreign-born status, whereas NFS workers did.

When FS groups were stratified by sociodemographic subgroups and compared with NFS counterparts, higher percentages of FMF and FBS workers who were NH-White, Hispanic, ages 30–39, male, female, above poverty <US$75k, insured, not foreign-born, with comorbidities or without comorbidities were considered reachable. Finally, there were additional significant differences among those reachable in each FS group (Table A1).

### Unvaccinated, Reluctant

Higher percentages of AFFH workers (26.3%) were reluctant to get vaccinated compared with NFS workers (18.2%). When comparing workers in the same worker group, FS and NFS workers had consistently significantly different rates of reluctance by race/ethnicity (lower rates of reluctant Hispanic workers compared with NH-White), age (lower rates of reluctant ages 50–64 compared with ages 18–29), and language of interview (lower rates of reluctant Spanish interview compared with English interview). Reluctance did not significantly differ by insurance status for FS workers, whereas it did for NFS workers. When FS groups were stratified by sociodemographic subgroups and compared with NFS counterparts, higher percentages were reluctant of AFFH workers who were NH-White; male; ages 18–29, 30–39, or 50–64; above poverty <US$75k or ≥US$75k; insured; not foreign-born; without comorbidities; interviewed in English; and non-principal city MSA residents. Finally, there were additional significant differences among those reluctant in each FS group (Table A1).

### Attitudes and Perceptions of COVID-19 and the COVID-19 Vaccine

**Figure 1.**
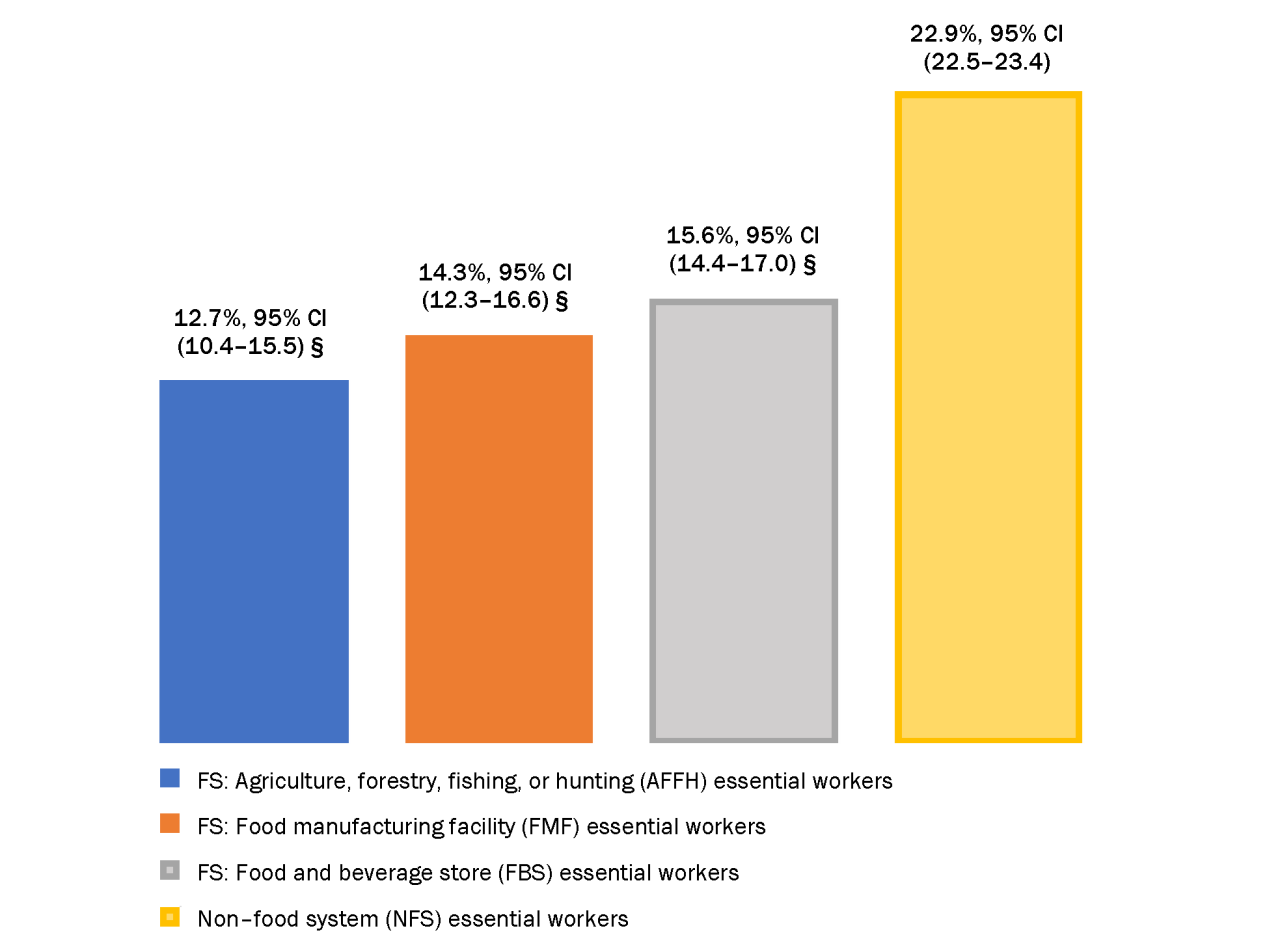
Percentage (%) of Essential Workers Reporting Work or School COVID-19 Vaccine Requirements, National Immunization Survey Adult COVID Module, April 22–November 27, 2021 § Statistically significant at *p* < 0.05 compared with the referent group (differences compared with non–food system workers).

Compared with NFS workers, significantly lower overall proportions of FS workers reported concern about getting COVID-19; significantly lower proportions of AFFH and FMF workers think the vaccine is important for protection; and lower percentages of AFFH workers think that the vaccine is safe. There were large differences in concern about getting COVID-19, confidence that the vaccine is safe, and in attitudes about its importance for protection, within FS worker groups and between FS and NFS worker groups by race/ethnicity, sex, age, household income, insurance, foreign-born status, language of interview, comorbidity status, and urbanicity (Table A1). Significantly lower percentages of FS workers reported that work or school require the vaccine compared with NFS essential workers (Figure 1).

### Experiences and Difficulties Getting the Vaccine

Overall, fewer than 10% of vaccinated FS workers reported difficulties knowing where to get vaccinated, how to get to vaccination sites, and whether vaccination sites were open at convenient times. Less than 20% of vaccinated workers reported difficulty getting the vaccine or getting an appointment online—all of which were not statistically different from NFS workers. A significantly higher proportion of unvaccinated AFFH and FBS workers compared with vaccinated counterparts reported that it was hard to get to vaccination sites, or that sites were not open at convenient times. Significantly lower proportions of unvaccinated FMF and FBS workers compared to vaccinated counterparts reported difficulties getting an appointment online; a higher proportion of unvaccinated FBS workers reported difficulties getting an appointment online or getting to vaccination sites compared with NFS workers (Table 2).

Tables B1–B3 offer a summary of statistically significant results of Tables A1–A3; Table B4 provides results overall and by month that were summarized in Table 2. These tables are found in Appendix B.

## Discussion

The results show that from May to November 2021—a period that included primary and booster shot availability, a summer SARS-CoV-2 Delta variant surge, and the onset of the new Omicron variant^10^—significantly lower percentages of FS workers (AFFH, FMF, FBS) reported being vaccinated with ≥1 dose when compared with NFS essential workers. This could be related to results that showed significantly lower proportions of FS workers that reported concern about getting COVID-19, or stronger work/school COVID-19 vaccine requirements compared with NFS workers. Less than 20% of FS and NFS workers reported vaccine difficulties, but with differences by work group and vaccination status. More research may be needed to understand what factors affected the differences in vaccine uptake between FS and NFS workers. Recovery from a past COVID-19 infection or variation in prioritizing and distributing vaccinations for frontline/essential worker groups could explain some results for FS workers compared to NFS workers (Johnson, 2021; Lutrick et al., 2022, National Academy for State Health Policy, 2021).^11^ Fewer reports of work/school requirements among FS workers may be explained by the NFS worker group including healthcare workers, who are more likely to be subject to workplace COVID-19 vaccination requirements.^12^ Finally, a number of other overlapping vulnerabilities such as occupation or work setting, which we discuss below in the context of results that were statistically significant, could have influenced results for FS workers. Overall results for uptake and demand, and work-related vaccine mandates, are consistent with other studies during this time period; however, they are not directly comparable due to differing industry/occupation groupings (Henneberger et al., 2022; King et al., 2021; Nguyen et al., 2021; Steege et al., 2022).

Stratifying worker groups by sociodemographic characteristics to compare outcomes between population subgroups of the same worker group revealed some similarities between FS and NFS workers, including lower percentages of uninsured FS and NFS workers receiving at least one dose. These similarities could suggest that some vaccine disparities by sociodemographic characteristics in our sample were not necessarily related to specific types of essential work. Many of these results among essential worker population subgroups, which are consistent with other sociodemographic data from this period, highlight how sociodemographic identities may be more broadly linked with certain disparities that stretch beyond occupation, essential worker status, or industry (CDC, 2023a, 2023b, 2023c). When we stratified worker groups by sociodemographic characteristics to compare with NFS counterparts, there was also some evidence to suggest that some results for FS worker subgroups were connected to specific workgroups. All three FS groups [AFFH, FMF, FBS] compared to NFS counterparts had significantly lower proportions of workers overall reporting uptake of ≥1 dose, particularly those who were NH-White, aged 30–39, insured, not foreign-born, or without comorbidities. However, compared to NFS counterparts, significantly higher percentages of FMF and FBS workers overall, and from the same sociodemographic subgroups, were unvaccinated and reachable, while AFFH workers were more reluctant.

### Individual FS Worker Groups (AFFH, FMF, FBS)

Results for the three individual FS worker groups are discussed below.

### Agriculture, Forestry, Fishing, or Hunting (AFFH) Workers

Overall, AFFH workers compared with NFS workers had less uptake and more reluctance to get vaccinated. We also found that a significantly smaller proportion reported concern about getting COVID-19, or belief that the COVID-19 vaccine is safe or important for protection. These results may have been influenced by work setting characteristics that could affect perceived risk of getting COVID-19 and importance of the vaccine, such as work that is mostly performed outside, in rural and remote locations, and away from the general public. Further, a larger proportion of unvaccinated AFFH workers reported difficulties getting to vaccination sites, or sites not being open at convenient times, compared to vaccinated AFFH workers. Some work-related factors could have influenced vaccine access in different work sectors and settings across the AFFH workforce. For example, fishing industry/sector AFFH workers may have challenges with vaccine access related to working offshore, on a boat, for extended periods of time, whereas other sectors/industries of AFFH may not be as remote.

Some AFFH findings were notable despite low uptake for the group overall, such as high uptake for AFFH workers with comorbidities (75.7% vs no comorbidities 55.3%). Those with any comorbidity had higher coverage in all groups, but we noted greater than 20 percentage points difference by comorbidity status for AFFH workers whereas the differences for other groups were more modest (percentage points difference for other groups ranged from 5.9% to 12.7%). Understanding these findings could suggest future areas of research that would inform interventions to improve adult infectious disease vaccine confidence and demand in vulnerable populations. Women are underrepresented in the AFFH workforce, and vulnerable populations such as people with comorbidities are overrepresented; AFFH workers were a population of focus for significant public and private efforts during the pandemic to address known overlapping vulnerabilities, and to close disparities in trusted, convenient, and linguistically and culturally appropriate ways (Corwin et al., 2021; Flynn, Eggerth et al., 2021; Flynn, Rodriguez Lainz et al., 2021; Hahn & Yiannas, 2021; Marcom et al., 2020; National Center for Farmworker Health, 2022; Stebbins & Pellizzari, 2021).

### Food Manufacturing Facility (FMF) Workers

When compared with NFS workers, significantly lower proportions of FMF workers overall reported vaccine uptake, concern about getting COVID-19, and the sentiment that the vaccine is important for protection, but a higher proportion of FMF workers were unvaccinated and considered reachable. Early experiences with COVID-19 could have impacted vaccine attitudes and behaviors for these workers; for example, changes in risk perception related to recovery from past COVID-19 infection, or work setting characteristics such as shift work schedules and rural/remote work locations that limited access to vaccine sites (Corkery & Yaffe-Bellany, 2020; Douglas, 2020, 2021; Parks et al., 2020).

### Food and Beverage Store (FBS) Workers

FBS workers reported significantly less coverage and concern about getting COVID-19 than NFS workers, but FBS workers compared with NFS workers were overall more likely to be unvaccinated and reachable, and overall had more favorable beliefs about the vaccine. As with other worker groups, FBS job functions and characteristics could have influenced overall results. These characteristics include having been classified as essential workers during the pandemic and the resulting public moralization of their work, regular exposure or interaction with the general public in work settings where customers remove protective masks to eat/drink, vaccine messaging through onsite pharmacies, and workplace vaccine mandates (Cameron et al., 2022; Mayer et al., 2022).

Compared with unvaccinated NFS workers, higher percentages of unvaccinated FBS workers reported difficulty getting online appointments and accessing vaccination sites, and reported sites not being open at convenient times compared to vaccinated FBS workers; however, a lower percentage of unvaccinated FBS workers reported difficulty getting online appointment compared with vaccinated FBS. Although some FBS workers may have more regular access to vaccine sites and exposure to vaccine messaging (for example, through grocery store pharmacies), this may not be the case for other FBS workers. The food and beverage industry, including the FBS group in this study, is composed of a wide variety of sectors and workers (e.g., grocery store cashier, waiter/waitress, food delivery driver) with largely varied work roles and responsibilities that may shape uptake, attitudes, and experiences. Schneider and colleagues (2021), analyzing vaccine uptake and intent among workers in various FBS sectors, found that 68% of service sector workers were vaccinated by November 2021. but that rates were lowest among large food service employers and widely variable between grocery sector employers (60%–86%).

### Strengths and Limitations

Strengths of this study include being the first national-level representative study to assess and characterize differences of self-reported COVID-19 vaccination coverage, intent, attitudes, and experiences among three different types of FS workers, and between FS and NFS workers, to determine if disparities exist. We used cross-sectional data from a large survey of U.S. adults conducted monthly and made available in languages other than English. The large overall sample size allowed for analysis of FS workers and stratification by sociodemographic characteristics. Finally, we differ from other studies using NIS-ACM data in that we are the first to analyze essential worker respondents grouped in the “other” category. Using NIOCCS to further identify FS and NFS workers from NIS-ACM data allowed us to analyze over 19,000 additional respondents in our sample who otherwise would have been excluded. In doing so, we were able to expand our analysis to include FS worker jobs that otherwise would not have been included in the original FS industry/occupation worker groups from the survey, such as food delivery drivers and online grocery order shoppers. Additionally, updated versions of the NIS-ACM survey now include expanded examples for the food system worker group industry/occupation classifications.^13^ NIOCCS is an accessible way to refine and incorporate occupational information into research and practice that is free, and easy to learn and use overall.

Results are subject to several limitations. First, COVID-19 vaccination was self-reported and might be subject to recall or social desirability bias. Second, our study captured data from May to November 2021 and may not reflect attitudes or experiences beyond this time period. Third, the response rate for NIS-ACM was low (<25%) although similar to those in other NIS surveys.^14^ Although data were weighted to reduce possible bias from incomplete sample frame or non-response and were calibrated to the COVID-19 vaccine administration data reported by jurisdictions to the CDC, bias might still persist and may impact generalizability of results from this study. Fourth, relatively small sample sizes for some sociodemographic groups may have resulted in low statistical power to detect differences by socio-demographics in stratified analysis.

## Conclusion

Results from our study demonstrated that, compared to NFS workers in May–November 2021, significantly lower proportions of FS workers (AFFH, FMF, FBS) overall were vaccinated with ≥1 dose. Less than 20% of vaccinated and unvaccinated FS and NFS workers and NFS counterparts reported vaccine difficulties, with differences by worker group and by vaccination status. Some disparities between certain FS worker sociodemographic subgroups were also found between the same NFS subgroups. Differences in attitudes and perceptions by occupational identity and sociodemographic characteristics were also noted. Nonetheless, our study shows that many disparities in vaccine uptake and intent existed between FS and NFS workers, and between workers in the same group. Results reflect the diversity of food system work and its workforce. Considering preservation of the functioning of essential businesses that supply food to the population during emergency and non-emergency times, and contribute to the health protection of communities and individuals, our findings present implications for both research and practice.

### Implications for Research

It may be important to collect and analyze occupational data and key demographic indicators—individually and in combination—to identify social determinants that could contribute to specific health inequities. Identifying these overlapping vulnerabilities may allow for a strategic tailoring of public health interventions, health-promotion systems, and infrastructure to address health inequities more effectively. Equitable vaccination for infectious diseases, such as COVID-19, is an important tool for closing persistent disparities, including preventing excess morbidity and mortality (Wong et al., 2021).

As previously noted, our study is perhaps the first to explore these outcomes in a representative national sample and with a specific emphasis on FS workers. The novelty of this research alone underscores the need for attention to and support for FS workers, given the vast literature examining these and further outcomes among other essential workers during the pandemic. Future research can further investigate work-related inequities, and could explore and refine our results with more advanced statistical methods or in the context of the unmeasured factors this study did not assess. These could include specific work sectors, the impacts of occupational exceptionalism, certain policies known to impact these workers (such as free COVID-19 vaccines for everyone regardless of immigration or health insurance status), or assessing these outcomes in relation to work-specific risk perception (for example, lower perceived risk of COVID-19 because of work mostly performed outdoors and away from the public during the pandemic). We contribute a rich sociodemographic and occupational dataset for three groups of essential FS workers during the COVID-19 pandemic, and direction for more refined analyses of these topics and populations in future research, interventions for adult infectious disease vaccination, and programmatic and policy aspects of future emergency management. Data and tools used in this study, such as NIOCCs and NIS-ACM data,^15^ are free, publicly accessible, and can be used to fill data gaps about FS workers and more broadly, support inclusion of work into research and programs.

### Implications for Practice

These results show opportunities for practitioners and organizations to find effective ways of reaching workers with vaccine and health information and interventions, and providing institutional support. Public health institutions can build and enhance collaborative partnerships with trusted organizations working to improve health outcomes in populations that have been marginalized. Providing funding, training, and technical assistance to build capacity of trusted organizations can help expand the reach and impact of the shared priorities of improving health and addressing disparities (e.g., improving vaccine uptake).

Trusted organizations supporting FS workers, and that are familiar with the community and specific to the occupational landscape, can help decrease intervention costs and improve the chances of adoption, implementation, and maintenance of interventions. Not only will priority populations be more likely to consider the trusted source credible, but these organizations can leverage existing assets and infrastructure to support interventions. For example, tailoring activities to occupational and workforce characteristics, such as developing and delivering messaging in linguistically and culturally appropriate ways, prioritizing and distributing vaccines in ways that consider the remote workplace nature and unique schedules that some FS workers face (e.g., working off-shore for long periods of time), delivering programs/interventions in familiar and convenient places (e.g., at worksites), and connecting workers to key community services.

Improved data collection and interpretation can help inform these efforts. For instance, practitioners and trusted organizations can collect and assess FS worker data to better document and characterize needs and barriers of these workers. Further, involvement with comprehensive planning for future emergencies that consider occupation-related barriers and health disparities can help sustain health promotion efforts, such as to take part in emergency response planning processes to help identify areas of greatest need, elevate FS worker considerations in certain emergency cases, and develop and deliver messaging in linguistically and culturally appropriate ways.

## Figures and Tables

**Table 1.** Results of Free-Text Analysis, Additional Essential Workers Categorized in Final Essential Worker Groups

Essential Worker Groups	Number of distinct free-text responses added to final sample^a^
Food System (FS) Essential Worker: Agriculture, forestry, fishing, or hunting (AFFH)	227
FS Essential Worker: Food manufacturing facility (FMF)	100
FS Essential Worker: Food and beverage store (FBS)	844
Non-food system Essential Workers (NFS)	18,293

a Distinct, free-text responses from 19,464 essential worker respondents who answered “Other” to survey question “In what location or setting do you currently work?”; many of the distinct free-text responses had multiple respondents.

**Table 2.** Overall Experiences and Difficulties with Getting the COVID-19 Vaccine among Food System (FS) and Non–Food System (NFS) Essential Workers, by Worker Vaccination Status, National Immunization Survey Adult COVID Module, April 22–November 27, 2021

	Agriculture, Forestry, Fishing, or Hunting (AFFH)	Food Manufacturing Facility (FMF)	Food and Beverage Store (FBS)	Non–food system (NFS) ^g^ (§Ref)
	(*n* = 1,772 vaccinated; 288 unvaccinated)	(*n* = 2,440 vaccinated; 413 unvaccinated)	(*n* = 8,014 vaccinated; 1,470 unvaccinated)	(*n* = 106,001 vaccinated; 9,596 unvaccinated)

Vaccine Related Outcome	%^a^ (95% CI)	%^a^ (95% CI)	%^a^ (95% CI)	%^a^ (95% CI)

**Difficulty getting vaccinated** ^b^				

Vaccinated (≥1 dose) (¶ref)	14.1 (11.0–17.9)	15.5 (12.8–18.5)	13.4 (12.0–15.0)	13.8 (13.4–14.3)

Unvaccinated	17.9 (10.2–29.3)	12.5 (8.4–18.2)	16.6 (13.7–20.1)	13.7 (12.5–14.9)

**Difficulty getting an appointment online** ^c^				

Vaccinated (≥1 dose) (¶ref)	15.8 (12.7–19.4)	17.1 (14.5–20.0)	16.2 (14.7–17.8)	15.3 (14.9–15.8)

Unvaccinated	9.7 (5.6–16.3)	*5.8 (3.8–9.0)* ¶	*10.2 (7.9–13.0)* ¶§	*7.0 (6.2–8.0)* ¶

**Difficulty with not knowing where to get vaccinated** ^d^				

Vaccinated (≥1 dose) (¶ref)	7.5 (5.6–10.1)	6.7 (5.1–8.9)	8.2(7.1–9.4)	7.0 (6.7–7.3)

Unvaccinated	14.7 (7.9–25.7)	9.1 (6.1–13.5)	10.4 (8.2–13.1)	8.8 (7.9–9.8)

**Hard to get to vaccination sites** ^e^				

Vaccinated (≥1 dose) (¶ref)	3.5 (2.2–5.4)	3.9 (2.7–5.7)	5.8 (4.9–6.9)	4.4 (4.2–4.7)

Unvaccinated	*12.1 (6.3–22.1)* ¶	6.1 (3.8–9.7)	*11.6 (9.0–14.8)* ¶§	7.5 (6.6–8.5)

**Sites are not open at convenient times** ^f^				

Vaccinated (≥1 dose) (¶ref)	6.6 (4.5–9.4)	6.2 (4.7–8.1)	5.9(5.0–6.9)	5.9 (5.6–6.2)

Unvaccinated	*18.2 (10.3–30.1)* ¶	10.5 (6.8–15.9)	*14.1 (11.4–17.3)* ¶	12.2 (11.1–13.4)

a Weighted percents.

b Respondents who reported getting a vaccine is or would be “very difficult” or “somewhat difficult.”

c-f Vaccination status/intent was not a prerequisite for questions about experiences and difficulties, and respondents could answer regardless of vaccination status; respondents who answered “not at all difficult” to question listed in b were not asked these questions.

g “Non–food system essential workers” included healthcare, social service, preschool or daycare, K-12 school, other schools and instructional settings, first response, death care, correctional facility, non-food manufacturing facility, public transit, and U.S. Postal Service. NIS Adult COVID Module (NIS-ACM) Hard Copy Questionnaire: Q3/2021 (https://www.cdc.gov/vaccines/imz-managers/nis/downloads/NIS-ACM-Questionnaire-Q3-2021.pdf)

¶ Statistically significant at *p* < 0.05 difference between vaccinated and unvaccinated worker in the same group.

§ Statistically significant at *p* < 0.05 difference between FS worker and NFS counterpart.

## Footnotes

1 Exceptionalism is the perception or belief that a species, country, society, institution, movement, individual, or time period is exceptional. The term conveys, whether or not specified, that the referent is superior in some way. In the domain of occupation/work, exceptionalism reflects exemption of certain workers from social, labor, health, and safety policies and protections. For example, despite difficult working conditions, farmworkers in the United States are excluded from much federal-level labor protection that applies to most other workers (Rodman et. al., 2016).

2
https://www.cdc.gov/vaccines/imz-managers/nis/about.html

3
https://aapor.org/wp-content/uploads/2022/11/Standard-Definitions20169theditionfinal.pdf

4
https://www.cdc.gov/vaccines/imz-managers/nis/downloads/NIS-ACM-Questionnaire-Q3-2021.pdf

5 See ACIP 3: https://www.cdc.gov/vaccines/imz-managers/nis/downloads/NIS-ACM-Questionnaire-Q3-2021.pdf

6 See ACIP 2 for complete list of industry/occupation options: https://www.cdc.gov/vaccines/imz-managers/nis/downloads/NIS-ACM-Questionnaire-Q3-2021.pdf

7 NFS essential workers included several categories: healthcare, social service, preschool or daycare, K-12 school, other schools and instructional settings, first response, death care, correctional facility, non-food manufacturing facility, public transit, United States Postal Service; see ACIP 2 https://www.cdc.gov/vaccines/imz-managers/nis/downloads/NIS-ACM-Questionnaire-Q3-2021.pdf

8 Denoted in results tables with ¶.

9 Denoted in results tables with §

10 CDC Museum COVID-19 Timeline. https://www.cdc.gov/museum/timeline/covid19.html

11 Johns Hopkins University. State Vaccination Plans. https://coronavirus.jhu.edu/vaccines/vaccine-state-plans

12 Centers for Medicare & Medicaid Services (CMS), Medicare and Medicaid Programs, Omnibus COVID-19 Health Care Staff Vaccination. (*Federal Register*, 2021). https://www.federalregister.gov/documents/2021/11/05/2021-23831/medicare-and-medicaid-programs-omnibus-covid-19-health-care-staff-vaccination

13 This study used NIS-ACM data from Q3 of 2021 (https://www.cdc.gov/vaccines/imz-managers/nis/downloads/NIS-ACM-Questionnaire-Q3-2021.pdf); beginning with Q3 of 2022 (https://www.cdc.gov/vaccines/imz-managers/nis/downloads/NIS-ACM-Questionnaire-Q3-2022.pdf), the FBS option includes additional examples to help capture an expanded group of essential food workers such as food services, delivery, and distribution, that are not otherwise captured in the other FS group options.

14 CDC. National Immunization Surveys (NIS) Data, Tables, and Documentation. https://www.cdc.gov/vaccines/imz-managers/nis/data-tables.html

15 NIS-ACM data are publicly accessible in a controlled environment via the National Center for Health Statistics Research Data Center (RDC) at https://www.cdc.gov/rdc/index.htm

## Appendix A.

**Table A1.** Vaccination Status and Intent^b^ among Food System (FS) and Non–Food System (NFS) Essential Workers, by Sociodemographic Characteristics, National Immunization Survey Adult COVID Module, April 22–November 27, 2021

	Agriculture, forestry, fishing, or hunting (AFFH)				Food Manufacturing Facility (FMF)				Food and Beverage Store (FBS)				Non-food system (NFS) ^g^ (^§^ref)			
	
		Vaccinated^c^	Reachable^d^	Reluctant^e^		Vaccinated^c^	Reachable^d^	Reluctant^e^		Vaccinated^c^	Reachable^d^	Reluctant^e^		Vaccinated^c^	Reachable^d^	Reluctant^e^
							
	*n*	%^a^ (95% CI)			*n*	%^a^ (95% CI)			*n*	%^a^ (95% CI)			*n*	%^a^ (95% CI)		

**Overall**	2,708	58.5 (54.7–62.2) §	15.2 (12.6–18.2)	26.3 (23.0–29.9) §	3,484	59.8 (56.4–63.1) §	18.9 (16.5–21.6) §	21.3 (18.3–24.7)	11,156	61.6 (59.7–63.5)§	20.4 (19.0–22.0) §	17.9 (16.4–19.6)	133,913	68.5 (68.0–69.1)	13.3 (12.8–13.7)	18.2 (17.8–18.7)

**Race/Ethnicity** ^f^																

White, non-Hispanic (¶ref)	1,917	56.8 (52.4–61.1) §	13.1(10.5–16.2)	30.1 (26.0–34.5) §	2,060	60.9 (56.5–65.0) §	15.2 (12.6–18.3) §	23.9 (20.0–28.4)	6,326	60.3 (57.8–62.8) §	18.5 (16.6–20.5) §	21.2 (18.9–23.7)	80,604	67.9 (67.2–68.6)	11.1 (10.6–11.6)	21.0 (20.4–21.6)

Black, non-Hispanic	96	60.6 (42.0–76.5)	26.3(13.2–45.6)	13.2 (5.4–28.8) ¶	349	49.9 (39.5–60.3) §	26.4 (18.9–35.6) ¶	23.6 (13.9–37.2)	1,240	55.5 (50.2–60.6) §	26.6 (22.1–31.7) ¶§	17.9 (13.9–22.7)	17,024	67.1 (65.5–68.6)	19.4 (18.1–20.8) ¶	13.5 (12.4–14.7) ¶

Hispanic	394	64.1 (54.8–72.5)	18.3 (12.1–26.7)	17.6 (11.2–26.5) ¶	618	62.9 (55.5–69.7) §	25.5 (19.3–32.8) ¶§	11.7 (7.81–7.1) ¶	1,918	65.7 (61.5–69.6) ¶§	24.8 (21.3–28.8) ¶§	9.5 (7.2–12.5) ¶§	18,129	71.3 (69.8–72.6) ¶	16.1 (15.0–17.3) ¶	12.6 (11.6–13.7) ¶

Other	217	60.6 (45.5–73.9)	11.0 (3.52–9.6)	28.4 (17.4–42.7)	363	59.3 (45.5–71.8)	15.5 (9.2–24.9)	25.2 (13.5–42.0)	1,439	68.9 (63.6–73.7) ¶	15.0 (11.7–19.0)	16.2 (12.3–20.9) ¶	14,706	71.9 (70.0–73.7) ¶	11.9 (10.6–13.4)	16.2 (14.7–17.9) ¶

American Indian/Alaska Native	55	50.3 (30.6–69.8)	2.0 (0.5–7.5) ¶	47.7 (28.3–67.8)	44	45.7 (20.6–73.1)	12.5 (4.7–29.4)	41.8 (18.4–69.7)	148	36.8 (26.1–48.9) ¶§	31.6 (20.2–45.9) §	31.5 (18.7–48.1) ¶	1,764	53.6 (48.4–58.8) ¶	17.4 (13.6–22.0) ¶	29.0 (23.9–34.7) ¶

Asian	52	86.0 (66.5–95.0) ¶	5.7 (1.1–25.6) §	8.3 (2.5–24.5) ¶	124	91.8 (79.4–97.0) ¶	7.4 (2.4–20.3)	0.8 (0.2–3.4)	605	94.0 (89.9–96.5) ¶	5.2 (2.8–9.3)	0.9 (0.4–1.9) §	6,010	91.1 (89.1–92.9) ¶	6.8 (5.2–8.8) ¶	2.1 (1.5–3.0) ¶

Native Hawaiian/Pacific Islander	<30	^h^	.	.	46	67.3 (40.0–86.5)	28.1 (10.8–56.0)	4.5 (0.7–24.2) ¶§	123	68.7 (50.6–82.4)	17.3 (8.8–31.1)	14.0 (5.6–30.9)	1,695	71.9 (65.6–77.5)	10.4 (7.4–14.4)	17.7 (13.0–23.6)

Multiple races/other	86	48.4 (27.6–69.7)	17.9 (4.7–49.1)	33.8 (16.2–57.4)	149	41.4 (24.1–61.1)	19.2 (8.7–37.3)	39.4 (19.4–63.8)	563	51.7 (43.6–59.7)	20.8 (14.9–28.2)	27.5 (20.4–36.0)	5,237	56.4 (53.2–59.5) ¶	16.1 (13.8–18.8) ¶	27.5 (24.5–30.7) ¶

**Sex**																

Male (¶ref)	2,081	54.4 (50.0–58.7) §	16.2 (13.1–19.9)	29.4 (25.4–33.7) §	2,299	59.2 (55.3–63.1)	19.8 (16.8–23.2) §	21.0 (17.7–24.7)	5,943	61.1 (58.5–63.6)	21.6 (19.6–23.8) §	17.3 (15.2–19.6) §	65,393	62.6 (61.8–63.4)	15.4 (14.7–16.0)	22.0 (21.3–22.7)

Female	601	73.7 (66.7–79.7) ¶	11.3 (7.4–16.8)	15.0 (10.4–21.2) ¶	1,148	59.9 (53.4–66.1) §	17.7 (13.9–22.3) §	22.3 (16.3–29.7) §	5,126	62.0 (59.2–64.7) §	19.3 (17.2–21.6) §	18.7 (16.4–21.3) §	67,587	74.8 (74.1–75.5) ¶	11.1 (10.6–11.7) ¶	14.1 (13.5–14.7) ¶

**Age**																

18–29 (¶ref)	509	37.7 (30.6–45.3) §	26.2 (18.8–35.2)	36.2 (28.4–44.7) §	878	49.1 (43.1–55.1)	24.9 (20.2–30.4)	26.0 (20.2–32.8)	3,840	54.5 (51.5–57.5)	26.6 (24.1–29.4) §	18.8 (16.3–21.6) §	24,579	52.2 (50.9–53.4)	22.3 (21.2–23.5)	25.5 (24.4–26.7)

30–39	543	51.9 (43.9–59.7) ¶§	17.4 (11.8–24.9)	30.7 (23.8–38.7) §	741	52.9 (45.4–60.3) §	23.2 (17.5–30.1) §	23.9 (17.4–31.9)	2,320	55.7 (51.8–59.6) §	24.0 (20.5–27.7) §	20.3 (17.2–23.8)	28,672	63.7 (62.5–64.8) ¶	15.4 (14.5–16.4) ¶	20.9 (19.9–22.0) ¶

40–49	498	62.1 (53.8–69.8) ¶	11.0 (6.9–17.0) ¶	26.9 (20.3–34.8)	640	63.8 (55.8–71.1) ¶	16.2 (11.7–21.8) ¶	20.1 (13.5–28.7)	1,762	63.1 (57.9–68.0) ¶§	17.1 (13.9–21.0) ¶§	19.8 (15.4–25.1)	27,499	69.3 (68.1–70.5) ¶	11.1 (10.3–12.0) ¶	19.6 (18.5–20.7) ¶

50–64	804	70.4 (63.4–76.6) ¶§	10.3 (7.3–14.3) ¶	19.3 (13.8–26.4) ¶§	1,026	70.6 (64.2–76.3) ¶§	12.8 (9.1–17.8) ¶§	16.5 (11.8–22.7) ¶	2,553	78.0 (74.4–81.2) ¶	8.7 (6.8–11.1) ¶	13.3 (10.6–16.5) ¶	41,657	80.5 (79.6–81.4) ¶	7.8 (7.2–8.4) ¶	11.7 (11.0–12.5) ¶

65+	316	81.2 (67.0–90.2) ¶	7.3 (3.5–14.3) ¶	11.5 (4.2–28.2) ¶	170	84.4 (70.2–92.5) ¶	5.9 (1.2–24.2) ¶	9.7 (4.6–19.5) ¶	559	87.0 (75.8–93.5) ¶	3.7 (1.6–8.4) ¶	9.2 (3.6–21.7) ¶	9,724	89.6 (88.0–91.1) ¶	4.7 (3.7–6.1) ¶	5.6 (4.6–6.8) ¶

**Household income**																

Below poverty	204	54.3 (41.1–66.9)	23.5 (13.3–38.1)	22.3 (13.5–34.4)	327	45.8 (35.4–56.6) ¶	31.8 (22.9–42.2) ¶	22.4 (12.6–36.6)	1,596	54.6 (49.9–59.2) ¶	27.2 (23.2–31.6) ¶	18.2 (14.6–22.4)	7,701	56.1 (53.8–58.4) ¶	23.6 (21.6–25.7) ¶	20.3 (18.4–22.3) ¶

Above poverty, <$75K	875	59.5 (53.1–65.6)	15.6 (11.5–20.8)	24.9 (19.6–31.0) §	1,361	54.2 (48.8–59.4) ¶§	21.6 (17.6–26.3) ¶§	24.2 (19.4–29.9) ¶§	4,763	58.9 (56.0–61.7) ¶§	22.2 (19.9–24.7) ¶§	18.9 (16.5–21.7)	42,703	65.0 (64.0–66.0) ¶	16.3 (15.5–17.1) ¶	18.7 (17.9–19.5) ¶

Above poverty, ≥$75K (¶ref)	1,060	60.3 (54.1–66.2) §	11.1 (8.1–15.2)	28.6 (23.0–34.9) §	1,133	72.8 (67.6–77.5)	11.8 (8.8–15.6)	15.4 (11.6–20.1)	2,530	72.3 (68.3–76.1)	11.1 (8.9–13.8)	16.5 (13.3–20.4)	59,519	74.9 (74.1–75.7)	8.8 (8.2–9.3)	16.3 (15.6–17.0)

Unknown income	569	56.6 (48.3–64.5) §	16.8 (11.4–24.0)	26.6 (19.8–34.7)	663	60.0 (52.1–67.5) ¶	16.4 (11.7–22.4)	23.6 (16.8–32.2)	2,267	60.7 (56.6–64.6) ¶§	22.0 (18.7–25.7) ¶§	17.3 (14.2–20.9)	23,990	65.8 (64.5–67.1) ¶	13.6 (12.6–14.6) ¶	20.6 (19.5–21.8) ¶

**Health insurance**																

Insured (¶ref)	2,246	62.4 (58.4–66.3) §	13.0 (10.5–15.9)	24.6 (21.2–28.4) §	2,941	62.7 (59.1–66.2) §	17.1 (14.6–19.9) §	20.2 (17.0–23.8)	8,960	65.3 (63.2–67.3) §	17.4 (15.8–19.0) §	17.3 (15.6–19.2)	120,919	70.9 (70.3–71.5)	12.1 (11.6–12.5)	17.0 (16.6–17.5)

Not insured	382	44.9 (35.6–54.5) ¶	21.1 (14.1–30.3)	34.1 (24.9–44.6)	449	43.5 (35.0–52.3) ¶	29.4 (22.0–38.0) ¶	27.2 (18.5–38.0)	1,838	47.1 (42.8–51.4) ¶	33.0 (28.9–37.3) ¶§	19.9 (16.4–24.0) §	9,847	48.7 (46.8–50.6) ¶	23.6 (21.8–25.4) ¶	27.8 (26.0–29.6) ¶

**Foreign born status**																

Foreign born	311	63.0 (51.4–73.3) §	21.6 (13.8–32.1)	15.4 (7.9–27.9) ¶	511	73.1 (64.7–80.2) ¶	21.9 (15.2–30.5)	4.9 (2.8–8.7) ¶	1,597	74.2 (69.2–78.7) ¶	20.5 (16.4–25.3) §	5.3 (3.3–8.4) ¶	17,331	78.2 (76.7–79.6) ¶	15.0 (13.7–16.3) ¶	6.9 (6.0–7.8) ¶

Not foreign born (¶ref)	2,258	57.8 (53.7–61.7) §	13.4 (10.9–16.4)	28.9 (25.2–32.8) §	2,799	57.0 (53.2–60.8) §	18.2 (15.7–21.1) §	24.7 (21.1–28.7) §	9,021	59.4 (57.4–61.5) §	20.4 (18.8–22.1) §	20.1 (18.3–22.1)	111,059	67.3 (66.7–67.9)	12.8 (12.4–13.3)	19.8 (19.3–20.4)

**Language of interview**																

English (¶ref)	2,545	58.1 (54.3–61.8) §	13.6 (11.3–16.4)	28.3 (24.8–32.0) §	3,351	59.3 (55.8–62.7) §	18.0 (15.7–20.6) §	22.7 (19.5–26.2) §	10,807	61.2 (59.3–63.1) §	20.1 (18.6–21.7) §	18.7 (17.1–20.4)	132,232	68.6 (68.0–69.1)	12.9 (12.5–13.3)	18.5 (18.1–19.0)

Spanish	160	61.2 (47.5–73.3)	23.8 (14.2–37.0)	15.0 (6.92–9.6) ¶	120	66.3 (48.5–80.5)	32.5 (18.5–50.6)	1.2 (0.3–4.1) ¶§	278	66.0 (55.5–75.1)	33.1 (24.0–43.7) ¶	0.9 (0.3–2.5) ¶§	1,407	64.2 (59.4–68.7)	31.2 (26.8–36.0) ¶	4.6 (3.1–6.9) ¶

Other	<30	.	.	.	--	.	.	.	71	98.1 (93.0–99.5) ¶§	1.3 (0.2–7.0) ¶§	0.5 (0.1–3.5) ¶	274	83.9 (74.9–90.1) ¶	11.9 (6.7–20.2)	4.2 (1.6–10.5) ¶

**Comorbidities**																

Yes (any)	552	75.7 (68.0–82.0) ¶	10.5 (6.3–16.8)	13.8 (9.1–20.4) ¶	788	64.9 (57.4–71.7) §	18.8 (13.7–25.3) §	16.3 (10.8–23.9)	2,621	71.1 (67.2–74.7) ¶§	14.9 (12.4–17.8) ¶§	14.0 (11.0–17.7) ¶	33,614	78.3 (77.2–79.2) ¶	9.8 (9.1–10.5) ¶	12.0 (11.2–12.8) ¶

No (¶ref)	2,124	55.3 (51.0–59.4) §	15.7 (12.8–19.2)	29.0 (25.2–33.2) §	2,666	59.0 (55.2–62.7) §	18.9 (16.2–21.9) §	22.1 (18.8–25.9)	8,405	59.1 (57.0–61.2) §	21.9 (20.2–23.8) §	19.0 (17.2–20.9)	99,157	65.6 (65.0–66.3)	14.2 (13.7–14.7)	20.1 (19.6–20.7)

**Urbanicity**																

MSA, principal city (¶ref)	443	66.7 (57.7–74.7)	13.9 (7.8–23.6)	19.4 (13.9–26.4)	1,009	66.7 (59.9–72.9)	15.7 (11.8–20.6)	17.6 (12.1–24.8)	3,621	65.9 (62.7–69.0) §	20.9 (18.2–23.8) §	13.2 (11.0–15.8)	40,761	72.7 (71.7–73.7)	13.4 (12.6–14.2)	13.9 (13.1–14.7)

MSA, non-principal city	1,132	60.5 (54.8–65.9) §	14.4 (10.9–18.8)	25.1 (20.2–30.7) §	1,661	60.2 (55.4–64.9) §	20.6 (17.2–24.5) §	19.2 (15.0–24.2)	5,298	62.5 (59.9–65.0) §	20.0 (18.0–22.1) §	17.5 (15.4–19.9) ¶	66,381	68.8 (68.0–69.5) ¶	13.2 (12.6–13.8)	18.0 (17.4–18.7) ¶

Non-MSA	1,133	50.0 (44.1–55.9) ¶§	17.3 (13.4–22.0)	32.7 (27.1–38.9) ¶	814	47.8 (41.1–54.6) ¶§	18.9 (14.0–25.2)	33.3 (27.2–39.9) ¶	2,237	49.4 (44.7–54.1) ¶§	21.3 (17.7–25.4) §	29.3 (24.6–34.4) ¶	26,771	58.8 (57.4–60.2) ¶	13.2 (12.2–14.3)	28.0 (26.7–29.3) ¶

**Month of interview**																

May (¶ref)	523	49.2 (41.3–57.1) §	17.9 (13.0–24.1)	32.9 (25.7–41.0) §	703	47.8 (41.2–54.5) §	28.4 (22.5–35.1) §	23.8 (18.6–29.9)	2,076	49.2 (45.2–53.3) §	34.2 (30.3–38.4) §	16.6 (13.6–20.0) §	22,650	58.3 (57.0–59.7)	20.3 (19.1–21.5)	21.4 (20.2–22.6)

June	350	53.0 (42.5–63.3)	15.9 (9.5–25.5)	31.1 (21.7–42.4)	471	49.8 (39.9–59.8) §	18.4 (12.7–26.0) ¶	31.7 (21.4–44.2)	1,392	57.1 (51.8–62.2) ¶	25.8 (21.3–30.8) ¶§	17.2 (13.3–22.0)	16,587	61.0 (59.4–62.6) ¶	18.0 (16.6–19.5) ¶	21.0 (19.7–22.4)

July	434	58.1 (48.7–66.8)	20.7 (13.4–30.6)	21.2 (15.0–29.0) ¶	548	58.5 (49.9–66.6)	19.1 (13.6–26.1) ¶	22.4 (15.1–31.9)	1,809	57.2 (52.4–61.9) ¶§	21.1 (17.3–25.5) ¶§	21.7 (17.7–26.4)	21,447	63.9 (62.5–65.3) ¶	15.1 (14.1–16.3) ¶	21.0 (19.7–22.3)

August	393	62.0 (53.2–70.1) ¶	14.3 (9.4–21.3)	23.7 (16.8–32.3)	457	71.2 (63.0–78.3) ¶	11.5 (7.3–17.7) ¶	17.2 (11.4–25.2)	1,501	65.0 (60.3–69.4) ¶	18.8 (15.3–22.8) ¶§	16.2 (12.8–20.4)	18,335	68.3 (66.9–69.7) ¶	13.4 (12.3–14.5) ¶	18.3 (17.2–19.6) ¶

September	374	64.2 (54.1–73.1) ¶	14.5 (8.5–23.6)	21.3 (14.6–30.1) ¶	518	63.1 (54.5–71.0) ¶§	19.3 (12.9–27.8) §	17.6 (11.8–25.4)	1,677	63.8 (59.1–68.2) ¶§	17.1 (13.9–20.8) ¶§	19.1 (15.2–23.8)	20,636	73.0 (71.8–74.3) ¶	11.2 (10.3–12.2) ¶	15.7 (14.7–16.8) ¶

October	438	71.5 (62.2–79.4) ¶	9.2 (5.4–15.2) ¶	19.3 (12.6–28.3) ¶	551	66.1 (58.6–72.8) ¶§	14.8 (10.2–21.0) ¶§	19.1 (13.8–25.8)	1,832	68.6 (63.6–73.2) ¶§	13.9 (10.8–17.8) ¶§	17.5 (13.5–22.2)	22,782	76.4 (75.1–77.6) ¶	8.0 (7.2–8.8) ¶	15.7 (14.6–16.8) ¶

November	196	59.4 (46.3–71.3) §	10.3 (4.0–23.9)	30.3 (19.8–43.4) §	236	72.5 (60.8–81.8) ¶	16.5 (9.3–27.8) ¶§	11.0 (5.6–20.2) ¶	869	73.0 (66.5–78.6) ¶	10.0 (6.9–14.2) ¶	17.1 (12.1–23.5)	11,476	79.2 (77.5–80.8) ¶	6.6 (5.6–7.8) ¶	14.2 (12.9–15.7) ¶

a Weighted percents.

b Vaccination status and intent is among those who answered both the vaccination question (Have you received at least one dose of a COVID-19 vaccine?) and the intent question (How likely are you to get a COVID-19 vaccine? Would you say you would definitely get a vaccine, probably get a vaccine, probably not get a vaccine, definitely not get a vaccine, or are not sure?).

c Respondents who self-reported having at least one dose of a COVID-19 vaccine.

d Respondents who self-reported that they “Definitely plan to get vaccinated” or “Probably will get vaccinated or unsure.’”

e Respondents who self-reported that they “Probably or definitely will not get vaccinated.”

f Race and ethnicity were assessed by the following two questions: “Are you of Hispanic or Latino origin?” and “Now, I am going to read a list of categories. Please choose one or more of the following categories to describe your race. Are you White, Black or African American, American Indian, Alaska Native, Asian, Native Hawaiian or other Pacific Islander?” Persons were categorized into mutually exclusive categories of race and ethnicity; persons who did not identify as Hispanic were categorized by their reported race or races.

g “Non-food system essential workers’ included healthcare, social service, preschool or daycare, K-12 school, other schools and instructional settings, first response, death care, correctional facility, non-food manufacturing facility public transit; and United States Postal Service NIS Adult COVID Module (NIS-ACM) Hard Copy Questionnaire: Q3/2021 (cdc.gov)

h Cells with denominator n <30 are suppressed.

¶ Statistically significant at p <0.05 compared with the referent group (Differences within worker group).

§ Statistically significant at p <0.05 compared with the referent group (Differences compared with non-food system workers).

**Table A2.** Attitudes and Perceptions of COVID-19 Vaccine Uptake among Food System (FS) and Non-Food System (NFS) Essential Workers, by Sociodemographic Characteristics, National Immunization Survey Adult COVID Module, April 22–November 27, 2021

	Agriculture, forestry, fishing, or hunting (AFFH)				Food Manufacturing Facility (FMF)				Food and Beverage Store (FBS)				Non–Food System (NFS) ^e^ (§ref)			
	*n*	Concerned about getting COVID-19^b^	Thinks a COVID-19 vaccine is safe^c^	Thinks a COVID-19 vaccine is important protection^d^	*n*	Concerned about getting COVID-19^b^	Thinks a COVID-19 vaccine is safe^c^	Thinks a COVID-19 vaccine is important protection^d^	*n*	Concerned about getting COVID-19^b^	Thinks a COVID-19 vaccine is safe^c^	Thinks a COVID-19 vaccine is important protection^d^	*n*	Concerned about getting COVID-19^b^	Thinks a COVID-19 vaccine is safe^c^	Thinks a COVID-19 vaccine is important protection^d^
	%^a^ (95% CI)				%^a^ (95% CI)				%^a^ (95% CI)				%^a^ (95% CI)			
**Overall**	2,730	29.9 (26.6–33.4) §	49.8 (46.0–53.6) §	67.4 (63.8–70.8) §	3,495	35.7 (32.7–38.7) §	55.4 (52.1–58.7)	72.0 (68.6–75.2) §	11,189	37.0 (35.3–38.8) §	56.5 (54.5–58.4)	77.2 (75.4–78.8)	134,375	39.0 (38.5–39.5)	57.5 (56.9–58.1)	75.6 (75.0–76.1)
**Race/Ethnicity**																
White, non-Hispanic (¶ref)	1,926	25.0 (21.5–28.8) §	50.6 (46.1–55.1) §	61.1 (56.6–65.3) §	2,064	28.3 (24.9–31.9) §	56.6 (52.4–60.8)	70.7 (66.6–74.5)	6,344	30.5 (28.4–32.6) §	58.7 (56.1–61.3)	73.4 (70.9–75.8)	80,835	33.0 (32.3–33.6)	59.9 (59.2–60.7)	72.1 (71.4–72.7)
Black, non-Hispanic	97	55.4 (36.6–72.7) ¶	42.3 (26.1–60.3)	75.1 (50.6–89.9)	349	50.9 (40.4–61.4) ¶	52.1 (42.7–61.3)	70.0 (57.4–80.2) §	1,242	44.4 (39.4–49.6) ¶ §	46.3 (40.9–51.8) ¶	77.7 (72.7–82.0)	17,057	54.4 (52.9–55.9) ¶	50.1 (48.5–51.7) ¶	82.4 (81.0–83.7) ¶
Hispanic	396	38.5 (30.4–47.3) ¶	50.4 (41.6–59.3)	86.0 (79.2–90.8) ¶	619	44.7 (37.7–51.8) ¶	56.3 (48.8–63.4)	80.9 (73.6–86.5) ¶	1,925	47.2 (43.1–51.3) ¶	55.4 (51.1–59.6)	86.3 (83.1–89.0) ¶ §	18,172	44.8 (43.3–46.2) ¶	57.2 (55.7–58.7) ¶	82.3 (81.1–83.4) ¶
Other	221	32.4 (20.2–47.6)	52.2 (37.6–66.3)	60.2 (46.0–72.8) §	366	41.8 (30.6–53.9) ¶	50.3 (37.1–63.4)	61.7 (47.1–74.4) §	1,443	45.1 (39.9–50.4) ¶	58.9 (53.3–64.2)	79.9 (74.8–84.2) ¶	14,770	45.8 (44.0–47.6) ¶	56.9 (54.9–58.8) ¶	78.8 (77.1–80.4) ¶
American Indian/Alaska Native	56	15.9 (7.8–29.8) §	38.4 (20.8–59.7)	46.1 (27.2–66.1)	45	32.5 (12.0–63.0)	22.9 (8.8–47.9) ¶	29.8 (13.9–52.9) ¶ §	150	36.0 (24.0–49.9)	46.8 (33.5–60.5)	65.3 (50.0–77.9)	1,774	38.3 (33.6–43.1) ¶	43.3 (38.6–48.1) ¶	63.1 (57.8–68.1) ¶
Asian	52	43.9 (20.8–69.9)	84.3 (68.0–93.1) ¶ §	80.4 (56.1–92.9) ¶	124	67.1 (50.3–80.5) ¶	77.3 (60.1–88.5) ¶	98.3 (95.2–99.4) ¶ §	605	55.4 (47.0–63.4) ¶	71.9 (64.1–78.6) ¶	95.6 (86.4–98.7) ¶	6,012	56.3 (53.5–59.0) ¶	71.0 (68.4–73.6) ¶	94.8 (93.3–95.9) ¶
Native Hawaiian/Pacific Islander	<30	^f^	--	--	46	32.4 (13.5–59.5)	68.5 (39.3–87.9)	71.8 (43.2–89.5)	123	34.1 (19.4–52.7) §	56.5 (36.6–74.4)	81.0 (62.9–91.5)	1,702	53.8 (47.1–60.3) ¶	46.5 (39.7–53.4) ¶	80.6 (75.1–85.1) ¶
Multiple races/other	89	32.5 (14.9–57.0)	39.7 (21.0–61.9)	54.1 (32.7–74.1)	151	30.2 (16.2–49.1)	36.5 (19.9–57.1)	46.3 (27.3–66.5) ¶	565	38.3 (30.6–46.7)	48.2 (39.9–56.7) ¶	68.1 (59.7–75.5)	5,282	35.6 (32.8–38.5)	46.2 (43.1–49.4) ¶	65.7 (62.5–68.7) ¶
**Sex**																
Male (¶ref)	2,099	26.4 (22.7–30.3) §	48.2 (43.8–52.6) §	63.6 (59.3–67.7) §	2,309	33.3 (29.8–37.0)	57.0 (53.1–60.8)	72.4 (68.7–75.8)	5,961	33.3 (31.1–35.6)	59.1 (56.5–61.7) §	76.6 (74.2–78.9) §	65,671	32.2 (31.5–33.0)	55.0 (54.1–55.8)	69.9 (69.1–70.7)
Female	602	42.7 (35.6–50.1) ¶	56.2 (48.4–63.7)	81.7 (75.6–86.6) ¶	1,148	41.0 (35.4–46.8) ¶	52.2 (45.9–58.3) §	71.9 (64.7–78.2) §	5,140	41.1 (38.5–43.7) ¶ §	53.3 (50.5–56.1) ¶ §	77.7 (75.0–80.1) §	67,727	46.1 (45.3–46.8) ¶	60.3 (59.5–61.1) ¶	81.7 (81.1–82.4) ¶
**Age**																
18–29 =(¶ref)	512	22.0 (16.1–29.2) §	34.6 (27.5–42.3)	59.3 (51.1–67.0) §	878	29.8 (24.7–35.4)	47.4 (41.6–53.2)	64.2 (57.5–70.5)	3,848	32.9 (30.3–35.7) §	56.9 (53.8–59.8) §	76.2 (73.3–78.8) §	24,636	28.7 (27.7–29.8)	48.0 (46.8–49.3)	67.7 (66.5–68.9)
30–39	547	28.6 (21.7–36.8) §	44.4 (36.4–52.7) §	61.8 (53.8–69.2) §	746	34.5 (27.7–42.0)	52.1 (44.9–59.1)	70.8 (64.1–76.8)	2,328	35.2 (31.6–38.9)	51.2 (47.2–55.2) ¶	74.8 (71.1–78.1)	28,757	37.7 (36.6–38.8) ¶	54.3 (53.1–55.5) ¶	72.4 (71.3–73.5) ¶
40–49	500	30.1 (23.0–38.2) §	45.7 (37.0–54.8) §	65.0 (56.3–72.8) §	642	40.7 (33.9–47.9) ¶	55.5 (47.3–63.3)	75.1 (66.5–82.2) ¶	1,772	39.2 (34.7–43.9) ¶	52.0 (46.7–57.2)	73.6 (68.1–78.4)	27,601	40.8 (39.6–42.0) ¶	56.8 (55.5–58.0) ¶	74.2 (73.0–75.3) ¶
50–64	808	36.9 (30.4–43.8) ¶ §	60.6 (53.2–67.6) ¶	75.7 (68.7–81.6) ¶ §	1,027	40.6 (35.0–46.5) ¶	64.5 (57.8–70.7) ¶	77.6 (71.0–83.0) ¶	2,558	46.4 (42.5–50.3) ¶	63.1 (59.0–67.0) ¶	83.8 (80.5–86.6) ¶	41,781	45.1 (44.0–46.1) ¶	64.7 (63.7–65.7) ¶	82.5 (81.6–83.3) ¶
65+	319	33.1 (23.1–44.9) §	76.9 (67.1–84.4) ¶	84.0 (75.0–90.2) ¶	172	30.2 (20.1–42.6) §	73.9 (59.4–84.6) ¶	87.3 (76.8–93.4) ¶	559	42.1 (34.0–50.7) ¶	67.9 (57.9–76.4) ¶	88.7 (77.0–94.9) ¶	9,751	47.2 (45.0–49.4) ¶	74.6 (72.6–76.6) ¶	90.8 (89.4–92.0) ¶
**Household income**																
Below poverty	204	48.7 (35.9–61.7) ¶	48.3 (34.9–61.9)	77.5 (65.5–86.2)	327	34.4 (25.2–44.9)	49.8 (40.0–59.6) ¶	77.5 (69.1–84.2)	1,603	40.8 (36.3–45.3)	48.4 (43.7–53.2) ¶	78.5 (74.4–82.1)	7,741	42.2 (40.1–44.4) ¶	46.2 (43.9–48.5) ¶	74.9 (72.8–76.9) ¶
Above poverty, <$75K	880	31.4 (26.0–37.4) §	49.3 (42.9–55.8)	69.1 (62.9–74.7)	1,366	35.0 (30.3–39.9)	50.0 (44.7–55.2) ¶	67.8 (62.0–73.1) ¶ §	4,775	37.4 (34.8–40.0)	54.8 (51.8–57.7) ¶	76.4 (73.6–79.1)	42,799	39.5 (38.6–40.4)	54.6 (53.6–55.6) ¶	75.1 (74.2–76.0) ¶
Above poverty, ≥$75K (¶ref)	1,064	27.7 (22.8–33.1) §	56.3 (50.1–62.3) §	67.5 (61.5–73.0) §	1,137	35.4 (30.4–40.8)	67.3 (61.7–72.4)	77.7 (72.7–82.1)	2,537	34.9 (31.4–38.7)	67.2 (63.1–71.0)	78.8 (74.8–82.3)	59,647	38.7 (38.0–39.5)	65.0 (64.2–65.9)	77.9 (77.2–78.7)
Unknown income	582	22.9 (17.3–29.8) §	41.1 (33.3–49.4) ¶ §	60.1 (52.1–67.6) §	665	38.2 (31.5–45.4)	51.1 (43.1–59.1) ¶	68.7 (60.1–76.2)	2,274	35.8 (32.2–39.5)	53.6 (49.4–57.7) ¶	75.8 (71.9–79.3) §	24,188	37.4 (36.2–38.6)	50.3 (48.9–51.6) ¶	71.6 (70.4–72.9) ¶
**Health insurance**																
Insured (¶ref)	2,260	30.2 (26.7–33.9) §	51.6 (47.5–55.8) §	66.9 (63.0–70.7) §	2,949	36.3 (33.1–39.6)	56.7 (53.2–60.1)	73.3 (69.8–76.6)	8,983	37.8 (35.9–39.8)	58.5 (56.3–60.6)	78.1 (76.1–80.0)	121,290	39.7 (39.1–40.2)	59.3 (58.7–60.0)	76.8 (76.3–77.3)
Not insured	386	29.4 (21.1–39.4)	44.9 (35.1–55.0)	69.9 (60.6–77.9)	452	31.8 (24.3–40.3)	48.8 (39.0–58.7)	66.2 (55.8–75.3)	1,845	34.4 (30.5–38.6)	49.0 (44.5–53.5) ¶ §	73.6 (69.4–77.4) §	9,897	34.3 (32.5–36.1) ¶	43.2 (41.3–45.2) ¶	66.3 (64.4–68.1) ¶
**Foreign born status**																
Foreign born	313	44.1 (33.9–54.9) ¶	55.1 (44.4–65.4)	92.3 (87.8–95.2) ¶ §	512	50.6 (42.5–58.8) ¶	63.6 (55.1–71.3) ¶	88.7 (81.6–93.3) ¶	1,600	47.4 (42.6–52.3) ¶	61.7 (56.6–66.6) ¶	89.5 (85.6–92.4) ¶	17,361	50.2 (48.6–51.8) ¶	63.0 (61.4–64.6) ¶	87.8 (86.6–88.9) ¶
Not foreign born (¶ref)	2,274	27.8 (24.4–31.5) §	49.4 (45.3–53.6) §	62.2 (58.1–66.1) §	2,808	33.1 (29.9–36.5) §	53.9 (50.2–57.6)	68.7 (64.8–72.3) §	9,048	35.2 (33.4–37.1) §	55.8 (53.7–58.0)	75.1 (73.1–77.0)	111,443	37.4 (36.9–38.0)	57.2 (56.6–57.9)	74.0 (73.4–74.5)
**Language of interview**																
English (¶ref)	2,565	29.3 (26.1–32.9) §	50.4 (46.4–54.3) §	62.9 (59.0–66.5) §	3,361	35.1 (32.1–38.2) §	55.1 (51.7–58.5)	70.9 (67.4–74.1) §	10,837	37.1 (35.4–38.9) §	56.2 (54.3–58.2)	76.5 (74.6–78.2)	132,679	39.1 (38.5–39.6)	57.6 (57.0–58.1)	75.3 (74.8–75.8)
Spanish	162	32.7 (22.0–45.6)	45.9 (33.5–58.8)	93.3 (86.8–96.7) ¶ §	121	43.3 (28.4–59.5)	63.1 (46.6–77.0)	88.1 (70.7–95.8) ¶	281	35.4 (26.3–45.7)	62.4 (52.3–71.6)	91.8 (85.0–95.7) §	1,420	31.7 (27.6–36.3) ¶	53.9 (48.9–58.9)	85.1 (81.0–88.4) ¶
Other	<30	--	--	--	<30	--	--	--	71	37.5 (21.7–56.5)	57.0 (34.8–76.7)	100.0 (99.8–100.0) §	276	58.4 (47.0–69.1) ¶	56.4 (44.5–67.5)	96.8 (93.4–98.5) ¶
**Comorbidities**																
Yes (any)	553	41.9 (34.3–49.9) ¶ §	60.4 (52.1–68.2) ¶	77.5 (69.6–83.8) ¶	790	45.8 (39.2–52.5) ¶	60.2 (52.6–67.4)	77.1 (69.2–83.5)	2,627	52.4 (48.5–56.2) ¶	59.7 (55.6–63.6)	83.9 (80.2–87.0) ¶	33,678	52.5 (51.4–53.6) ¶	63.0 (61.8–64.1) ¶	84.3 (83.4–85.2) ¶
No (¶ref)	2,142	27.3 (23.7–31.3) §	47.8 (43.5–52.1) §	65.4 (61.4–69.3) §	2,674	33.0 (29.7–36.5)	54.4 (50.8–58.1)	71.0 (67.3–74.4)	8,429	32.8 (31.0–34.8)	55.6 (53.4–57.8)	75.4 (73.4–77.4) §	99,484	34.7 (34.1–35.3)	55.9 (55.2–56.6)	73.0 (72.3–73.6)
**Urbanicity**																
MSA, principal city (¶ref)	451	38.2 (30.1–47.1)	59.8 (50.9–68.1)	75.8 (68.5–81.9)	1,012	43.6 (37.6–49.9)	56.9 (50.1–63.4)	74.8 (67.6–80.8)	3,629	41.1 (38.1–44.2)	58.6 (55.4–61.8)	81.6 (78.7–84.2)	40,890	43.5 (42.5–44.5)	61.0 (60.0–62.1)	80.7 (79.8–81.5)
MSA, non-principal city	1,140	29.8 (25.1–35.0) §	47.5 (42.0–53.2) ¶ §	69.0 (63.4–74.1) §	1,666	36.1 (32.0–40.4)	58.9 (54.5–63.2)	74.4 (69.7–78.5)	5,313	36.0 (33.7–38.4) ¶	58.1 (55.4–60.7)	77.7 (75.2–80.0) ¶	66,601	38.2 (37.5–39.0) ¶	57.8 (57.0–58.6) ¶	75.6 (74.9–76.3) ¶
Non-MSA	1,139	24.7 (20.2–29.8) ¶ §	47.0 (41.0–53.1) ¶	59.0 (53.2–64.6) ¶	817	22.3 (17.9–27.3) ¶ §	43.1 (35.9–50.5) ¶	61.1 (54.1–67.7) ¶	2,247	32.1 (28.1–36.5) ¶	45.9 (41.0–50.9) ¶	65.9 (60.7–70.7) ¶	26,884	32.6 (31.3–33.8) ¶	48.8 (47.3–50.2) ¶	64.8 (63.4–66.1) ¶
**Month of interview**																
May (¶ref)	525	30.8 (23.9–38.7)	42.6 (34.8–50.8) §	64.4 (56.1–71.9) §	704	32.4 (26.4–38.9)	54.4 (47.3–61.3)	71.4 (64.7–77.2)	2,081	37.9 (34.0–42.0) §	54.2 (49.9–58.5)	79.2 (75.5–82.4) §	22,715	533.5 (32.3–34.8)	55.7 (54.3–57.0)	74.4 (73.1–75.6)
June	353	22.5 (14.7–32.9)	48.6 (38.3–59.0)	70.9 (61.2–79.1)	473	24.0 (17.4–32.1)	52.8 (43.5–62.0)	68.9 (57.7–78.3)	1,399	29.4 (24.9–34.2) ¶	57.8 (52.3–63.1)	78.0 (73.0–82.3)	16,624	29.3 (27.9–30.8) ¶	55.6 (53.9–57.2)	73.7 (72.2–75.2)
July	436	28.8 (20.8–38.4)	49.2 (39.9–58.6)	75.7 (68.4–81.8) ¶	551	33.5 (26.4–41.4)	53.2 (44.4–61.7)	70.4 (61.0–78.3)	1,813	34.3 (30.1–38.8)	53.7 (48.8–58.5)	77.0 (72.6–80.9)	21,519	33.5 (32.2–34.7)	55.3 (53.9–56.7)	74.0 (72.7–75.3)
August	399	27.9 (21.0–35.9) §	54.6 (45.7–63.2)	64.1 (55.3–72.1) §	460	41.8 (34.2–49.8)	60.4 (52.0–68.3)	77.4 (69.2–84.0)	1,504	37.7 (33.6–42.1) §	54.2 (49.4–58.9)	77.9 (73.3–81.8)	18,401	44.3 (42.9–45.7) ¶	55.1 (53.6–56.5)	76.2 (74.9–77.4)
September	375	41.2 (31.4–51.7)	53.5 (43.0–63.6)	63.8 (53.5–73.0) §	520	43.3 (35.9–51.1) ¶	54.5 (46.1–62.7)	73.6 (64.5–81.0)	1,683	43.2 (38.9–47.6)	53.6 (48.9–58.2) §	76.2 (71.5–80.3)	20,715	47.0 (45.7–48.3) ¶	58.8 (57.4–60.1) ¶	76.6 (75.4–77.8) ¶
October	445	41.0 (31.8–50.9)	56.7 (46.1–66.7) ¶	66.1 (55.8–75.1) §	551	36.4 (29.8–43.5) §	52.4 (44.7–60.0) §	68.3 (60.9–75.0) §	1,839	40.4 (36.2–44.8)	61.8 (57.0–66.4) ¶	74.5 (69.4–78.9)	22,876	44.3 (43.0–45.6) ¶	61.1 (59.7–62.5) ¶	77.3 (76.1–78.5) ¶
November	197	23.4 (15.8–33.3) §	46.7 (34.6–59.2) §	64.6 (51.5–75.8)	236	45.3 (34.1–57.0)	62.6 (50.2–73.5)	76.5 (64.2–85.5)	870	36.6 (31.0–42.5)	60.5 (54.0–66.6)	77.3 (70.7–82.8)	11,525	41.3 (39.5–43.1) ¶	60.9 (59.0–62.7) ¶	76.8 (75.0–78.4) ¶

a Weighted percents.

b Respondents who answered “very concerned” or “moderately concerned” about getting COVID-19

c Respondents who reported that the COVID-19 vaccine is “completely safe” or “very safe”

d Respondents who reported the COVID-19 vaccine is “very important” or “somewhat important” to protect yourself against COVID-19

e “Non-food system essential workers” included healthcare, social service, preschool or daycare, K-12 school, other schools and instructional settings, first response, death care, correctional facility, non-food manufacturing facility, public transit, and United States Postal Service. NIS Adult COVID Module (NIS-ACM) Hard Copy Questionnaire: Q3/2021 (cdc.gov)

b-d Vaccination status/intent was not a prerequisite for questions about attitudes, and respondents could answer regardless of vaccination status

f Cells with denominator *n* < 30 are suppressed.

¶ Statistically significant at *p* < 0.05 compared with the referent group (Differences within worker group)

§ Statistically significant at *p* < 0.05 compared with the referent group (differences compared with non-food system workers).

## Appendix B.

**Table B1.** At-a-Glance of Statistically Significant Differences: Overall % of Food System (FS) Workers Reporting Vaccine Uptake, Intent, Attitudes/Beliefs, and Experiences, Compared with Non–Food System (NFS) Workers, National Immunization Survey Adult COVID Module, April 22–November 27, 2021

Vaccine Outcome and Direction of Finding	AFFH	FMF	FBS
Lower percentage (%) workers vaccinated with ≥1 dose	X	X	X
Higher % workers reachable		X	X
Higher % workers reluctant	X		
Lower % workers report concern about getting COVID-19	X	X	X
Lower % workers report the vaccine is important for protection	X	X	
Lower % workers report the vaccine is safe	X		
Higher % unvaccinated workers report trouble getting appointment online			X
Higher % unvaccinated workers report it is hard to get to vaccination sites			X

**Table B2.** At-a-glance of Statistically Significant Differences: Similarities between Food System (FS) and Non-Food System (NFS) Worker Sociodemographic Subgroups for COVID-19 Vaccine Coverage and Intent, and Concern about Getting COVID-19, May-November 2021

Vaccine Outcomes	Vaccinated ≥1 Dose	Unvaccinated, Reachable	Unvaccinated, Reluctant	Concerned about getting COVID-19
**Worker Groups**		FS [AFFH, FMF, FBS] and NFS		
FS and NFS Sociodemographic Subgroups with Significant Differences Compared with Workers in the Same Group	Significantly higher percentages of these FS and NFS workers reported having ≥1 dose compared to same worker group reference: • Asian workers compared to NH-W • Workers aged 40–65+ compared with 18–29	Significantly higher percentages of these FS and NFS workers were considered reachable compared to same worker group reference: • Workers aged 40–65+ compared with 18–29	Significantly higher percentages of these FS and NFS workers were considered reluctant compared to same worker group reference: • Workers residing in non-MSA compared with principal city MSA	Significantly higher percentages of these FS and NFS workers reported concern about getting COVID-19 compared to same worker group reference: • NH-B workers compared with NH-W • Hispanic workers compared with NH-W • Female workers compared with male • Workers aged 50–64 compared with 18–29 • Foreign born workers compared with non-foreign born • Workers with any comorbidities compared with none
	Significantly lower percentages of these FS and NFS workers reported having ≥1 dose compared to same worker group reference: • Uninsured workers compared with insured workers • Workers residing in non-MSA compared with principal city MSA		Significantly lower percentages of these FS and NFS workers were considered reluctant compared to same worker group reference: • Hispanic workers compared with NH-W • Workers aged 50–64 compared with 18–29 • Workers who had a Spanish interview compared with English interview	Significantly lower percentages of these FS and NFS workers reported concern about getting COVID-19 compared to same worker group reference: • Workers residing in non-MSA compared with principal city MSA

**Table B3.** At-a-Glance of Statistically Significant Differences: COVID-19 Vaccination Coverage and Intent, Overall and by Sociodemographic Characteristics, Food System (FS) Workers Compared with Non–Food System (NFS) Workers, May–November 2021

Vaccine Uptake and Demand Outcomes	Vaccinated ≥1 Dose	Unvaccinated, Reachable	Unvaccinated, Reluctant
**FS Worker Group(s)**	AFFH, FMF, FBS	FMF, FBS	AFFH
**Overall, FS worker groups compared with NFS**	Significantly **lower percent (%) of** AFFH, FMF, FBS workers were vaccinated with ≥1 dose	Significantly **higher % of** FMF or FBS workers were unvaccinated and reachable	Significantly **higher % of** AFFH workers were unvaccinated and reluctant
**FS sociodemographic subgroups with significant differences compared with NFS** ^a^	*Lower % NH-W*	*Higher % NH-White*	*Higher %* NH-White
	*Lower % those 30–39 years*	Higher % Hispanic	Higher % male
	*Lower % insured*	*Higher % ages 30–39*	Higher % 18–29
	*Lower % non foreign born*	Higher % below poverty <75K	*Higher % 30–39*
	*Lower % without comorbidities*	Higher % male	Higher % 50–64
	Lower % residing in a non-principal city MSA	Higher % female	Higher % below poverty <75k
	Lower % residing in non-MSA	*Higher % insured*	Higher % above poverty ≥75k
		*Higher % not foreign born*	*Higher % insured*
		*Higher %* with comorbidities	*Higher % non foreign born*
		*Higher % without comorbidities*	*Higher % without comorbidities*
			Higher % reside in non-principal city MSA

a Results for uptake and reachable FS workers compared to NFS by sociodemographic groups are included if the significant finding for a subgroup was consistently noted for all worker groups described in corresponding overall results compared with NFS workers. Italicized subgroups in all three columns indicate those that were consistently noted for all three outcomes- uptake, reachable, and reluctant. for example, significantly lower percentages of FS workers were vaccinated compared with NFS workers, and significantly higher percentages of FMF and FBS workers were unvaccinated but reachable. Significantly lower percentages of NH-W FS workers were vaccinated, but significantly higher percentages of NH-W FMF and FBS workers were unvaccinated but reachable, whereas significantly higher percentages of NH-W AFFH workers were unvaccinated but reluctant.

**Table B4.** Overall Experiences and Difficulties with Getting the COVID-19 Vaccine among Food System (FS) and Non–Food System (NFS) Essential Workers, by Vaccination Status and Month of First Vaccination, or Month of Interview, National Immunization Survey Adult COVID Module, April 22–November 27, 2021

	Agriculture, Forestry, Fishing, or Hunting (AFFH)						Food Manufacturing Facility (FMF)						Food and Beverage Store (FBS)						Non-Food System ^d^ (§Ref)					
	*n*	Difficulty getting vaccinated ^b^	Difficulty getting an appointment online ^c^	Difficulty with not knowing where to get ^bd^vaccinated	Hard to get to vaccination sites ^e^	Sites are not open at convenient times ^f^	*n*	Difficulty getting vaccinated ^b^	Difficulty getting an appointment online ^c^	Difficulty with not knowing where to get ^bd^vaccinated	Hard to get to vaccination sites ^e^	Sites are not open at convenient times ^f^	*n*	Difficulty getting vaccinated ^b^	Difficulty getting an appointment online ^c^	Difficulty with not knowing where to get ^bd^vaccinated	Hard to get to vaccination sites ^e^	Sites are not open at convenient times ^f^	*n*	Difficulty getting vaccinated ^b^	Difficulty getting an appointment online ^c^	Difficulty with not knowing where to get ^bd^vaccinated	Hard to get to vaccination sites ^e^	Sites are not open at convenient times ^f^
		% (95% CI)						% (95% CI)						% (95% CI)						% (95% CI)				
**Month of First Vaccine, Vaccinated (≥1 dose)**																								
Overall (¶ref)	1,772	14.1 (11.0–17.9)	15.8 (12.7–19.4)	7.5 (5.6–10.1)	3.5 (2.2–5.4)	6.6 (4.5–9.4)	2,440	15.5 (12.8–18.5)	17.1 (14.5–20.0)	6.7 (5.1–8.9)	3.9 (2.7–5.7)	6.2 (4.7–8.1)	8,014	13.4 (12.0–15.0)	16.2 (14.7–17.8)	8.2 (7.1–9.4)	5.8 (4.9–6.9)	5.9 (5.0–6.9)	106,001	13.8 (13.4–14.3)	15.3 (14.9–15.8)	7.0 (6.7–7.3)	4.4 (4.2–4.7)	5.9 (5.6–6.2)
On or before Dec 2020	<30	.	.	.	.	.	<30	.	.	.	.	.	44	10.4 (2.8–31.8)	4.2 (1.1–14.7)	0.0 (.-.)§	2.2 (0.5–10.1)	0.9 (0.1–7.1)	10,995	4.4 (3.5–5.7)	3.7 (3.0–4.5)	1.7 (1.4–2.2)	1.3 (1.0–1.7)	2.1 (1.7–2.7)
Jan 2021	84	14.8 (5.8–33.2)	5.5 (2.4–11.8) §	4.0 (1.3–11.5)	3.3 (1.1–9.4)	2.0 (0.5–8.1)	63	14.9 (4.9–37.4)	7.5 (2.9–17.9)	4.4 (1.0–17.4)	0.2 (0.0–1.5) §	3.8 (0.7–18.0)	229	(5.7–16.9)	9.8 (5.5–16.7)	3.4 (1.6–6.8) §	4.9 (2.6–9.2)	5.6 (2.6–11.6)	17,135	10.6 (9.7–11.6)	12.7 (11.7–13.7)	6.0 (5.3–6.8)	3.6 (3.1–4.2)	4.7 (4.1–5.4)
Feb 2021	188	8.9 (4.8–15.9) §	17.6 (8.5–32.9)	6.9 (2.7–16.4)	6.7 (1.4–27.2)	10.7 (3.4–28.6)	191	29.4 (18.5–43.3) §	30.1 (19.2–43.8)	6.8 (3.0–15.0)	2.3 (0.6–9.0)	6.6 (2.7–15.6)	573	18.4 (13.5–24.5)	16.6 (12.1–22.4)	10.2 (6.8–15.0)	7.7 (4.7–12.4)	7.0 (4.3–11.1)	15,555	16.4 (15.2–17.5)	18.7 (17.5–19.9)	8.0 (7.2–8.9)	5.0 (4.4–5.7)	6.0 (5.3–6.8)
Mar 2021	591	18.5 (12.7–26.2)	21.1 (15.2–28.6)	10.0 (6.6–14.9)	4.2 (2.4–7.3)	6.6 (3.5–12.3)	806	15.3 (11.6–19.9)	19.9 (15.5–25.2)	7.3 (4.7–11.4)	4.6 (2.2–9.1)	6.9 (4.3–10.9)	2,224	15.5 (12.8–18.7)	23.4 (20.0–27.3)	9.6 (7.2–12.8)	6.5 (5.0–8.3)	5.1 (3.9–6.6) §	25,126	18.5 (17.5–19.5)	22.0 (20.9–23.0)	9.3 (8.6–10.1)	5.8 (5.2–6.4)	7.3 (6.6–8.1)
Apr 2021	485	15.2 (9.6–23.2)	14.9 (10.2–21.3)	8.6 (4.8–14.9)	3.4 (1.8–6.4) §	6.9 (3.6–12.9)	714	17.4 (11.6–25.2)	19.2 (14.0–25.6)	9.1 (5.5–14.9)	5.1 (2.9–8.6)	8.2 (4.9–13.5)	2,504	15.6 (13.0–18.7)	20.1 (17.1–23.3)	9.9 (7.9–12.3)	7.5 (5.5–10.1)	8.2 (6.1–10.8)	19,033	16.8 (15.8–18.0)	20.1 (19.0–21.3)	9.4 (8.5–10.3)	5.8 (5.1–6.7)	6.9 (6.2–7.7)
May 2021	199	8.9 (4.6–16.7)	12.9 (7.2–21.9)	4.7 (2.3–9.5)	0.6 (0.2–1.7) §	4.5 (1.9–10.2)	328	9.6 (5.3–16.8)	12.6 (7.4–20.7)	2.9 (1.6–5.2) §	1.6 (0.7–4.0) §	3.8 (1.9–7.3)	1,217	11.2 (7.5–16.3)	10.5 (8.0–13.8)	7.5 (5.0–11.1)	3.8 (2.5–5.9)	4.6 (3.0–7.0)	8,241	10.9 (9.6–12.4)	11.6 (10.3–13.0)	5.8 (4.9–6.8)	3.7 (3.0–4.6)	6.2 (5.2–7.3)
June 2021	65	6.3 (1.5–22.3)	4.7 (0.8–23.5)	1.1 (0.3–4.4) §	4.5 (0.9–20.3)	8.7 (2.42–6.8)	135	18.6 (10.2–31.5)	12.9 (5.4–27.8)	3.8 (1.4–10.1)	8.3 (2.3–25.5)	4.9 (2.0–11.7)	525	4.2 (2.7–6.5) §	5.8 (3.1–10.7)	5.2 (2.5–10.8)	5.0 (2.1–11.0)	2.6 (1.2–5.6) §	3,503	9.2 (7.5–11.2)	7.1 (5.4–9.1)	4.1 (3.0–5.6)	2.9 (1.9–4.4)	6.2 (4.7–8.2)
July 2021	47	0.0 (.-.)§	12.5 (3.0–39.9)	0.0 (.-.)§	0.5 (0.1–2.5) §	8.1 (1.1–40.1)	75	6.2 (1.8–19.5)	3.1 (0.8–11.7)	8.7 (1.4–39.8)	0.0 (0.0–0.3) §	0.9 (0.2–3.4) §	280	9.4 (4.7–17.8)	5.2 (2.2–11.6)	4.3 (1.4–12.0)	2.1 (0.6–7.4)	5.7 (2.5–12.8)	2,270	7.6 (6.1–9.5)	4.5 (3.3–6.0)	4.4 (3.1–6.1)	2.7 (1.8–4.1)	4.4 (3.3–5.9)
Aug 2021	51	21.2 (5.1–57.5)	2.2 (0.3–14.7)	0.0 (.-.)§	0.0 (.-.)§	1.4 (0.3–6.0) §	72	7.5 (2.8–18.8)	4.3 (1.2–13.7)	4.5 (1.3–14.0)	5.2 (0.9–24.0)	7.5 (2.8–18.9)	260	7.3 (4.0–13.0)	1.5 (0.8–2.8) §	1.9 (0.8–4.4)	2.9 (0.7–11.3)	4.7 (1.8–11.5)	2,471	10.4 (8.3–13.0)	4.6 (3.3–6.5)	2.9 (2.0–4.3)	2.0 (1.3–3.2)	4.3 (3.2–5.8)
Sept 2021	<30	.	.	.	.	.	32	6.6 (1.1–31.2)	0.4 (0.1–3.0) §	3.2 (0.4–20.4)	0.0 (.-.)§	0.0 (.-.)§	120	10.9 (4.8–22.9)	3.9 (1.1–12.4)	1.1 (0.2–5.3)	0.3 (0.1–1.1) §	2.3 (0.4–13.1)	1,264	10.4 (7.4–14.5)	2.9 (1.6–5.5)	1.9 (1.1–3.1)	1.4 (0.8–2.6)	4.3 (2.7–6.9)
Oct 2021	<30	.	.	.	.	.	<30	.	.	.	.	.	34	12.9 (4.7–30.5)	5.9 (1.2–25.1)	5.9 (1.2–25.1)	2.5 (0.3–16.4)	8.6 (2.4–26.8)	351	14.8 (9.4–22.6)	6.6 (3.2–13.1)	5.9 (2.7–12.5)	1.9 (0.8–4.7)	4.4 (1.7–11.2)
Nov 2021	<30	.	.	.	.	.	<30	.	.	.	.	.	<30	.	.	.	.	.	57	11.0 (4.9–22.5)	0.5 (0.1–3.4)	0.8 (0.1–5.4)	1.9 (0.4–7.7)	12.8 (2.7–43.4)
**Month of Interview, Unvaccinated**																								
Overall	288	17.9 (10.2–29.3)	9.7 (5.6–16.3)	14.7 (7.9–25.7)	12.1 (6.3–22.1) ¶	18.2 (10.3–30.1) ¶	413	12.5 (8.4–18.2)	5.8 (3.8–9.0) ¶	9.1 (6.1–13.5)	6.1 (3.8–9.7)	10.5 (6.8–15.9)	1,470	16.6 (13.7–20.1)	10.2 (7.9–13.0) ¶ §	10.4 (8.2–13.1)	11.6 (9.0–14.8) ¶ §	14.1 (11.4–17.3) ¶	9,596	13.7 (12.5–14.9)	7.0 (6.2–8.0) ¶	8.8 (7.9–9.8)	7.5 (6.6–8.5)	12.2 (11.1–13.4)
May 2021	77	13.0 (5.7–26.9)	8.4 (3.0–21.8)	12.5 (5.4–26.0)	7.9 (2.3–23.5)	8.8 (3.9–18.7)	133	9.7 (5.0–18.0) §	7.9 (3.8–15.8)	9.2 (4.6–17.5)	5.6 (2.6–11.9)	5.3 (2.6–10.6) §	470	18.1 (13.0–24.5)	14.3 (9.8–20.6)	13.9 (9.5–19.8)	13.3 (8.5–20.4)	14.2 (10.0–19.7)	2,717	16.6 (14.3–19.1)	10.8 (9.0–12.9)	12.8 (10.8–15.2)	8.9 (7.1–11.1)	15.0 (12.8–17.5)
June 2021	51	34.9 (12.4–67.1)	9.0 (2.7–26.4)	27.8 (7.3–65.2)	9.9 (2.3–34.0)	38.7 (14.9–69.4)	63	16.4 (5.3–40.9)	3.2 (1.1–9.3)	3.7 (1.4–9.6) §	2.9 (1.1–7.6) §	22.9 (10.0–44.2)	207	10.3 (5.5–18.7)	6.0 (2.3–14.9)	4.9 (2.0–11.3)	11.0 (5.7–20.2)	11.3 (6.5–19.0)	1,409	13.1 (10.4–16.3)	6.4 (4.5–9.0)	8.9 (6.6–11.7)	7.4 (5.4–9.9)	12.1 (9.5–15.3)
July 2021	47	14.3 (3.2–45.6)	10.6 (3.1–30.4)	9.2 (2.3–30.3)	25.7 (8.4–56.7)	18.0 (4.9–48.0)	57	12.9 (4.8–30.2)	8.4 (3.6–18.8)	9.7 (3.6–23.9)	9.2 (3.1–24.4)	8.7 (3.0–22.9)	214	19.3 (12.6–28.5)	7.2 (3.7–13.7)	12.4 (7.3–20.2)	9.4 (5.6–15.5)	13.6 (8.3–21.4)	1,553	11.9 (9.5–14.7)	5.7 (4.1–7.9)	6.6 (4.9–8.9)	6.5 (4.7–9.0)	10.0 (7.9–12.6)
Aug 2021	35	14.9 (3.8–43.7)	20.8 (7.2–47.1)	20.5 (7.0–47.0)	14.9 (3.9–43.3)	22.4 (7.9–49.4)	37	6.5 (1.8–21.3)	10.8 (2.2–39.0)	14.5 (3.8–42.3)	5.7 (0.8–31.2)	18.9 (6.2–45.3)	181	12.8 (6.5–23.5)	9.2 (4.3–18.5)	9.2 (5.2–15.8)	9.9 (4.7–19.4)	13.8 (7.6–23.7)	1,255	12.8 (9.8–16.5)	5.3 (3.5–8.0)	7.2 (5.1–10.0)	6.6 (4.5–9.6)	10.7 (8.2–13.9)
Sept 2021	30	6.7 (2.1–19.0)	1.8 (0.2–12.3)	0.6 (0.1–4.6) §	0.6 (0.1–4.6) §	0.6 (0.1–4.7) §	43	15.3 (5.3–37.2)	1.1 (0.2–5.8) §	4.7 (1.3–15.6)	4.6 (1.3–15.5)	9.9 (3.5–25.0)	176	17.3 (9.8–28.9)	6.2 (3.1–12.3)	9.2 (3.6–21.6)	10.3 (5.7–17.8)	14.7 (8.0–25.7)	1,209	14.5 (11.3–18.3)	5.9 (4.0–8.8)	8.8 (6.3–12.2)	7.6 (5.4–10.7)	11.4 (8.7–14.9)
Oct 2021	36	29.0 (9.6–61.3)	12.8 (1.9–52.8)	26.1 (7.5–60.6)	13.2 (2.0–52.7)	16.7 (3.7–51.0)	58	20.2 (8.9–39.8)	1.3 (0.4–4.9) §	18.4 (6.5–42.1)	6.8 (1.0–33.9)	8.2 (1.5–33.9)	160	17.1 (8.7–31.0)	10.6 (4.0–25.0)	11.9 (5.0–25.9)	5.1 (2.5–10.1)	12.2 (5.1–26.4)	1,043	15.6 (11.7–20.4)	8.7 (5.7–13.0)	8.2 (5.6–12.0)	9.0 (6.0–13.2)	14.6 (10.3–20.2)
Nov 2021	<30	.	.	.	.	.	<30	.	.	.	.	.	62	27.3 (12.7–49.4)	19.3 (8.1–39.4)	8.8 (2.7–25.2)	26.2 (11.2–49.9)	24.8 (10.8–47.2)	410	8.7 (4.9–15.0)	3.8 (1.9–7.4)	5.1 (2.8–9.0)	6.2 (2.8–13.3)	10.7 (6.3–17.5)

a Weighted percents.

b Respondents who reported getting a COVID-19 vaccine is or would be ‘very difficult’ or ‘somewhat difficult’

c-f Respondents could answer regardless of vaccination status; respondents who answered ‘not at all difficult’ to question listed in b were not asked this.

d “Non-food system essential workers” included: healthcare; social service; preschool or daycare; K-12 school; other schools and instructional settings; first response; death care; correctional facility; non-food manufacturing facility; public transit; and United States Postal Service; NIS Adult COVID Module (NIS-ACM) Hard Copy Questionnaire: Q3/2021: https://www.cdc.gov/vaccines/imz-managers/nis/downloads/NIS-ACM-Questionnaire-Q3-2021.pdf

Cells with denominator *n* < 30 are suppressed.

¶ Statistically significant at *p* < 0.05 difference between vaccinated and unvaccinated worker in the same group

§ Statistically significant at *p* < 0.05 difference between FS worker and NFS counterpart
